# Contribution of common and rare variants to bipolar disorder susceptibility in extended pedigrees from population isolates

**DOI:** 10.1038/s41398-020-0758-1

**Published:** 2020-02-24

**Authors:** Jae Hoon Sul, Susan K. Service, Alden Y. Huang, Vasily Ramensky, Sun-Goo Hwang, Terri M. Teshiba, YoungJun Park, Anil P. S. Ori, Zhongyang Zhang, Niamh Mullins, Loes M. Olde Loohuis, Scott C. Fears, Carmen Araya, Xinia Araya, Mitzi Spesny, Julio Bejarano, Margarita Ramirez, Gabriel Castrillón, Juliana Gomez-Makhinson, Maria C. Lopez, Gabriel Montoya, Claudia P. Montoya, Ileana Aldana, Javier I. Escobar, Jorge Ospina-Duque, Barbara Kremeyer, Gabriel Bedoya, Andres Ruiz-Linares, Rita M. Cantor, Julio Molina, Giovanni Coppola, Roel A. Ophoff, Gabriel Macaya, Carlos Lopez-Jaramillo, Victor Reus, Carrie E. Bearden, Chiara Sabatti, Nelson B. Freimer

**Affiliations:** 1 Department of Psychiatry and Biobehavioral Sciences, University of California, Los Angeles, Los Angeles, CA 90095 USA; 2 Center for Neurobehavioral Genetics, Semel Institute for Neuroscience and Human Behavior, University California Los Angeles, Los Angeles, CA USA; 3 Bioinformatics Interdepartmental Program, University of California, Los Angeles, Los Angeles, CA 90095 USA; 4 Federal State Institution “National Medical Research Center for Preventive Medicine” of the Ministry of Healthcare of the Russian Federation. Petroverigskiy lane 10, Moscow, 101990 Russia; 5 Department of Computer Science, University of California, Los Angeles, Los Angeles, CA 90095 USA; 6 Department of Genetics and Genomic Sciences, Icahn Institute for Genomics and Multiscale Biology, Icahn School of Medicine at Mount Sinai, New York, NY 10029 USA; 7 King’s College London, Social, Genetic and Developmental Psychiatry Centre, Institute of Psychiatry, Psychology and Neuroscience, De Crespigny Park, Denmark Hill, London, SE5 8AF UK; 8 Pamela Sklar Division of Psychiatric Genomics, Department of Genetics and Genomic Sciences, Icahn School of Medicine at Mount Sinai, New York, NY 10029 USA; 9 Cell and Molecular Biology Research Center, Universidad de Costa Rica, San Pedro de Montes de Oca, San José, 11501 Costa Rica; 10 Division of Pediatric Pulmonology, Hospital Nacional de Nin ~os, San Jose, Costa Rica; 11 Instituto de Alta Tecnologia Medica, Medellín, Antioquia, Colombia; 12 Department of Neuroradiology, Klinikum rechts der Isar, TUM, Munich, Germany; 13 Grupo de Investigación en Psiquiatría (Research Group in Psychiatry; GIPSI), Departamento de Psiquiatría Facultad de Medicina, Universidad de Antioquia, Medellín, 050011 Colombia; 14 Department of Psychiatry and Family Medicine, Rutgers-Robert Wood Johnson Medical School, Rutgers University, New Brunswick, NJ 08901 USA; 15 Department of Genetics, Evolution and Environment, University College London, London, WC1E 6BT UK; 16 Laboratory of Molecular Genetics, Institute of Biology, University of Antioquia, Medellín, 050010 Colombia; 17 Ministry of Education Key Laboratory of Contemporary Anthropology and Collaborative Innovation Center of Genetics and Development, Fudan University, Shanghai, 200438 China; 18 Aix Marseille Univ, CNRS, EFS, ADES, Marseille, France; 19 Department of Human Genetics, University of California Los Angeles, Los Angeles, CA 90095 USA; 20 BioCiencias Lab, 01010 Guatemala, Guatemala; 21 Department of Psychiatry, Brain Center Rudolf Magnus, University Medical Center Utrecht, Utrecht, Netherlands; 22 Mood Disorders Program, Hospital San Vicente Fundacion, Medellín, 050011 Colombia; 23 Department of Psychiatry and UCSF Weill Institute for Neurosciences, University of California, San Francisco, CA 94143 USA; 24 Department of Psychology, University of California, Los Angeles, Los Angeles, CA 90095 USA; 25 Department of Health Research and Policy, Division of Biostatistics, Stanford University, Stanford, CA 94305 USA

**Keywords:** Bipolar disorder, Comparative genomics

## Abstract

Current evidence from case/control studies indicates that genetic risk for psychiatric disorders derives primarily from numerous common variants, each with a small phenotypic impact. The literature describing apparent segregation of bipolar disorder (BP) in numerous multigenerational pedigrees suggests that, in such families, large-effect inherited variants might play a greater role. To identify roles of rare and common variants on BP, we conducted genetic analyses in 26 Colombia and Costa Rica pedigrees ascertained for bipolar disorder 1 (BP1), the most severe and heritable form of BP. In these pedigrees, we performed microarray SNP genotyping of 838 individuals and high-coverage whole-genome sequencing of 449 individuals. We compared polygenic risk scores (PRS), estimated using the latest BP1 genome-wide association study (GWAS) summary statistics, between BP1 individuals and related controls. We also evaluated whether BP1 individuals had a higher burden of rare deleterious single-nucleotide variants (SNVs) and rare copy number variants (CNVs) in a set of genes related to BP1. We found that compared with unaffected relatives, BP1 individuals had higher PRS estimated from BP1 GWAS statistics (*P* = 0.001 ~ 0.007) and displayed modest increase in burdens of rare deleterious SNVs (*P* = 0.047) and rare CNVs (*P* = 0.002 ~ 0.033) in genes related to BP1. We did not observe rare variants segregating in the pedigrees. These results suggest that small-to-moderate effect rare and common variants are more likely to contribute to BP1 risk in these extended pedigrees than a few large-effect rare variants.

## Introduction

Bipolar disorder (BP), consisting of episodes of mania and depression, has a heritability from twin studies estimated to be ~80%^1^. For BP, as for most other common disorders, SNP-based genome-wide association studies (GWAS) of large case/control samples have discovered many loci that contribute unequivocally to disease risk but that collectively explain only a small fraction of disease heritability. The most recent published BP GWAS, incorporating >20,000 cases and 30,000 controls, has reported 30 genome-wide significant SNP-associations and SNP-based heritability (*h*^2^_snp_) of 25% for BP1^2^. The hypothesis that rare single-nucleotide variants (SNVs) and rare copy number variants (CNVs) could explain a substantial proportion of the “missing heritability” of complex traits^3^ has motivated the rapid growth of whole-exome sequencing and whole-genome sequencing (WGS) throughout biomedicine, including psychiatry. More than for other psychiatric disorders, however, sequencing efforts to identify variants with a high impact on BP risk have continued to focus on pedigrees^4–7^. This focus reflects published descriptions, over several decades, of numerous extended families in which BP is observed across multiple generations; as would be expected if these pedigrees were segregating a relatively high-penetrance susceptibility variant.

Because the evidence in the literature regarding the apparent segregation of BP in extended pedigrees is mostly anecdotal^7–9^, we aimed to systematically characterize the genetic contribution to BP disease risk in a series of such families through evaluation of variants across the allele frequency spectrum. If rare variants contribute to this risk it is expected that they would be enriched in this sample, which is, to our knowledge, the largest BP pedigree sample sequenced to date. In addition, because a wide range of evidence indicates considerable etiological heterogeneity between BP1 and milder forms of BP^2,10^, this study focused exclusively on families ascertained for multiple individuals with BP1, a strategy that we reasoned would reduce the impact of such heterogeneity. In a further effort to reduce heterogeneity, we limited the data set to pedigrees derived from two Latin American populations that are considered closely related genetic isolates; the province of Antioquia in CO and the Central Valley of CR^11^.

We collected microarray SNP data for 838 family members (as reported previously)^12^, and performed high-coverage WGS on 449 individuals, selected because identity by descent information provided by them would enable imputation of rare variants in the family members who were not sequenced. We analyzed these data to obtain high-quality genotypes for SNVs and CNVs. With this information, we sought to evaluate the impact of both common and rare variants on BP1, focusing on two major questions about its genetic etiology. First, we attempted to evaluate the overall genetic architecture of BP1 in these families by characterizing the genome-wide burden of both common and rare genetic variation. For common variants, we calculated the genome-wide burden with polygenic risk scores (PRS), using the latest BP1 GWAS summary statistics^2^ and compared the polygenic burden of risk alleles in affected cases and related controls. For rare variants, the genome-wide burden contrasted the burden of rare variants predicted to be deleterious in a set of genes related to BP1 between affected cases and related controls. Second, we attempted to identify rare deleterious variants segregating in the families, using a new method that we developed for this purpose.

## Materials and methods

### Sample recruitment, microarray genotyping, and WGS

We recruited 26 pedigrees (15 from CR and 11 from CO), each ascertained for multiple individuals diagnosed with BP1 (Table 1). Some families were previously studied using linkage analysis^13–17^. The ascertainment and phenotyping strategy was previously reported^18^, and is briefly reviewed in the Supplementary Text. Control individuals were relatives of BP1 individuals in families, and either they went through the complete psychiatric evaluation and were found to have no mental illness, or they answered negatively to all Mini International Neuropsychiatric Interview^19^ questions related to mood or psychotic symptoms and were > 60 years of age. Individuals who were not diagnosed as BP1 or who were not considered as controls had unknown disease status. Written informed consent was obtained from all participants. Institutional Review Boards at participating institutions approved all study procedures. Using DNA extracted from whole blood we performed microarray genotyping using Illumina Omni 2.5 chips; as reported previously^12^ this procedure yielded data after QC for 838 individuals (206 BP1) with 2,026,257 SNPs (Supplementary Figure 1). For WGS, we used ExomePicks to identify the subset of individuals to sequence that would enable maximum opportunity to impute variants into the remaining genotyped pedigree members. Owing to budgetary constraints, 22 pedigrees out of the 26 pedigrees (449 individuals after QC) were sequenced including 143 BP1 (Supplementary Figure 1). Illumina performed WGS using HiSeq 2000 with 36× mean coverage (100 bp read length).

**Table 1:** Description of families included in the current study.

FamID	*N*	NBP	NControl	NMissing	NGeno	NWGS	NPheno	NMale
CO10	38	6	6	26	24	13	24	13
CO13	24	5	2	17	20	0	19	10
CO14	29	8	1	20	23	12	22	16
CO15	27	5	1	21	21	10	21	12
CO18	37	6	8	23	26	18	25	18
CO23	48	9	6	33	32	20	31	20
CO25	15	4	3	8	13	5	12	7
CO27	58	9	10	39	35	25	35	31
CO4	73	10	8	55	42	31	43	37
CO7	149	29	16	104	111	60	112	72
CO8	16	5	0	11	7	6	8	6
CR001	46	8	3	35	20	15	7	25
CR004	187	23	12	152	77	44	45	91
CR006	37	4	0	33	13	7	8	22
CR007	12	2	0	10	9	6	6	7
CR008	30	7	2	21	17	10	13	15
CR009	44	9	4	31	32	13	34	17
CR010	30	4	1	25	17	11	12	15
CR011	16	3	4	9	13	0	12	6
CR012	35	5	4	26	26	12	22	16
CR013	39	4	1	34	10	0	8	15
CR014	26	5	0	21	8	5	3	14
CR015	19	2	1	16	10	0	10	7
CR016	24	4	3	17	18	8	19	14
CR201	355	44	41	270	201	111	177	176
CR277	25	4	1	20	13	7	10	11
Total	1439	224	138	1077	838	449	738	693

*FamID* Family ID, *N* number of individuals in the family, *NBP* number of BP1 individuals in the family, *NControl* number of controls in the family, *NMissing* number of individuals with missing BP1 status in the family, *NGeno* number of genotyped family members after QC, *NWGS* number of sequenced family members after QC, *NPheno* number of family members with endophenotype data, *NMale* number of males in the family.

### Variant calling, QC, and imputation

We called SNVs using GATK best practices^20,21^. We removed variants that failed variant quality score recalibration and set each genotype whose quality score was ≤ 20 to missing (see Supplementary Text for details on QC). After QC, we had 449 individuals (143 BP1) and 20,396,290 SNVs (Supplementary Table 1). We then performed genotype refinement using Polymutt^22^, which corrected almost all Mendelian inheritance errors (Supplementary Table 2). To increase the sample size, we performed pedigree-aware genotype imputation using GIGI^23,24^, which imputed 334 individuals with only microarray data. After imputation, 782 individuals (190 BP1, 130 controls, and 462 unknown disease status) were either sequenced or imputed with high quality (see Supplementary Text and Supplementary Figure 2 for measuring imputation accuracy).

We performed genome-wide detection of CNVs using microarray and WGS data. For microarray data, we adapted a previously established pipeline^25^ based on PennCNV^26^, and QuantiSNP^27^. After removing individuals failing QC (Supplementary Figure 3), we detected 5,437 CNVs (3,317 deletions and 2,120 duplications) after filtering for rare events > 5 kb in length and spanned by a minimum of 10 probes among 782 individuals (189 BP1, 128 controls, and 465 unknown disease status). For WGS data, we called 8,768 bi-allelic deletions using GenomeSTRiP software^28,29^ among the 449 sequenced individuals, and used these calls to impute CNVs in the same set of individuals imputed for SNVs. We found that CNVs from GenomeSTRiP had low Mendelian error rate (Supplementary Figure 4) and low false-discovery rate (Supplementary Table 3 and Supplementary Text). A summary on the number of variants and individuals after QC and on which analysis is applied to each type of variant, is in Supplementary Table 4.

### Variant annotation

SNVs were mapped to UCSC knownGene^30^ and GENCODE V.19^31^ transcripts. To identify rare variants, we used both external and internal sources of allele frequency. For SNVs we used allele frequencies in 1000 Genomes^32^ (1KG) Colombians (CLM) and ExAC^33^ Latinos (AMR). For CNVs, we extracted frequency information from the Database of Genomic Variants Gold Standard Variants^34^ for microarray CNVs and from Phase 3 of 1KG^35^ (AMR) for WGS CNVs. If a variant in our data set is present in an external source, we considered it rare if its MAF is < 1% in that source. For variants not present in any external source, we considered them rare if their MAF is < 10% in our data set where MAF is estimated from all sequenced individuals. Deleterious SNVs are stop-gain/loss, splice-site, and missense variants predicted damaging by PolyPhen-2^36^.

### Estimation of global admixture proportions

We generated estimates of admixture proportions for the 838 individuals with microarray data using ADMIXTURE^37^ with 57,180 LD-pruned SNPs. The reference populations were CEU (*n* = 112) and YRI (*n* = 113) from HapMap^38,39^, and 52 Native American samples from Central or South America who have virtually no European or African admixture^40^. We compared the proportion of European ancestry between BP1 individuals and controls using both a linear mixed model (LMM) based on lmekin function in coxme R package^41^ and a generalized linear mixed model (GLMM) based on GMMAT software^42^; both took into account relatedness of individuals using a kinship matrix calculated from theoretical kinship. In LMM, the dependent variable was the proportion of European ancestry, whereas the independent variable was BP1 status, and it was vice versa in GLMM.

### PRS analysis

We calculated PRS of our samples with WGS data using PRSice^43^ and summary statistics from the Psychiatric Genomics Consortium (PGC) GWAS of BP1^2^ and schizophrenia (SCZ)^44^ after excluding A/T and G/C SNVs and SNVs in the MHC region. Our WGS data were LD clumped, and we retained from the GWAS summary statistics the most significant SNV for each clump. We used LMM and GLMM to test association between BP1 status and PRS at each of five GWAS *p* value thresholds while considering relationships among individuals and global admixture proportions of European ancestry. We used logistic regression without considering relationships to estimate Nagelkerke *R*^2^ as it was not straightforward to estimate *R*^2^ using GLMM. We also did not include the admixture proportions when calculating *R*^2^ because we were interested in variance of BP1 explained only by PRS.

### Identifying genes relevant to BP1

To increase power to detect effects of rare variants on BP1, we focused on genes for which a priori information indicated their relevance to BP1. To identify such genes, we utilized three sources of information. First, we performed a stratified LD score regression^45^ using the latest PGC BP1 GWAS summary statistics^2^ to identify cell-type specific promotor or enhancer regions in which BP1 heritability is enriched. Among the 10 cell-types groups tested, we observed enrichment of heritability for BP1 only in the central nervous system (CNS) group (Supplementary Figure 5), which contained 8,714 genes. Second, we used genes near 15 genome-wide significant independent lead SNPs in the latest PGC GWAS that analyzed only individuals with BP1, excluding individuals with other types of BP. We identified 72 genes around these SNPs using windows of 250 Kb. At last, we identified 99 genes within 1 Mb of BP1 linkage peaks (Supplementary Text, Supplementary Figure 6, Supplementary Table 5). These three sources yielded a gene-set of 8,757 unique protein-coding genes with one or more deleterious SNVs in at least one individual in our dataset (Supplementary Text and Supplementary Table 6).

### Rare variant burden analysis

We compared burden scores between BP1 individuals and controls. For SNVs, this score was the mean burden of rare deleterious SNVs in our gene-set, which corresponds to the fraction of deleterious alternative minor alleles at those SNVs that each individual has. For CNVs, it was the number of genes in our gene-set affected by rare CNVs. Individuals who carry an overall larger number of rare SNVs may carry a larger number of rare deleterious SNVs; we, therefore, also calculated mean burden of all rare SNVs in the gene-set. For CNVs, we calculated the total number of CNVs and the average size of all CNVs that each individual carried. The mean burden of rare deleterious SNVs was regressed on the mean burden of all rare SNVs using LMM to account for relatedness. We performed a similar correction for the CNV burden score using the total number of CNVs and the average size of CNVs, and for microarray CNVs we also corrected for genotyping batch. The residuals after the LMM were then quantile-normal (QN) transformed, and we compared the QN transformed residuals between BP1 individuals and controls using both LMM and GLMM while taking into account relationships among individuals and admixture proportions of European ancestry.

### Rare variant segregation analysis

Given a rare variant in a family, we developed a statistical approach that computes *p* values to estimate the probability of having the observed segregation pattern or more extreme segregation patterns under the null hypothesis of random segregation. Our segregation statistic for a rare variant (*S*_rare_) is the sum of the number of BP1 individuals with a rare allele and the number of controls without the allele. To calculate the *p* value of *S*_rare_, we assume that the rare allele was introduced by certain founders in a family (denoted as *F*^rv^), enumerate many random inheritance vectors (IVs), and find the proportion of IVs that generate the same or larger *S*_rare_ values (Supplementary Figure 7). To obtain a “Family-level” *p* value we computed the *p* value for each rare variant in each family. We also computed “Variant-level” and “Gene-level” *p* values that meta-analyzed *p* values using the Fisher’s method across different families and across different rare variants and families in a gene, respectively. The performance of our approach using simulation data and an approach to detect *F*^rv^ using imputation results are discussed in the Supplementary Text.

### Multiple testing correction

We summarized the types of variants (common or rare), the number of tests, and type of multiple testing correction applied to each analysis in Supplementary Table 7. There are two main questions of interest in this study: (1) identifying genetic architecture of BP1 and (2) identifying specific loci segregating in the CO/CR pedigrees. As expected, a majority of tests in this study were employed in addressing the second question where we implemented standard procedures to account for multiplicity. We did not perform multiple testing correction to analyses related to the first question, and while we can apply the study-wide testing correction that considers all tests performed in this study, it would inappropriately reduce our power to learn about genetic architecture if we treat *p* values from analyses related to genetic architecture as we would treat *p* values from the rare variant segregation analysis. As we perform 11 main analyses, we could use a significance threshold of 0.5/11 and account for further multiplicity within each main analysis. However, we opted against this idea as this is not standard and it would make it difficult to compare our results with those from other studies. Instead, we presented the total number of hypotheses tested and the multiplicity adjustment procedure in Supplementary Table 7. Furthermore, we did not use the term “significant” in describing our findings, we report *p* values explicitly to show the strength of evidence. At last, it is important to note that we report results of all our analyses, even when they do not lead to the identification of any promising hypothesis, thereby avoiding selection bias.

## Results

### Characteristics of admixture in the CO/CR Pedigrees

**Fig. 1:**
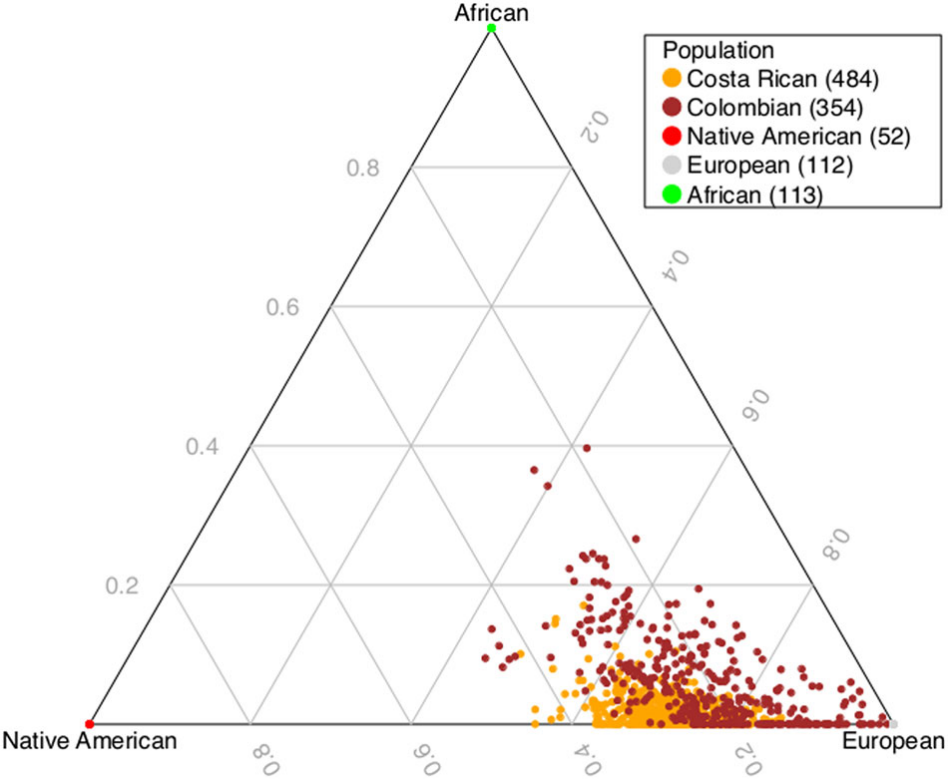
A de Finetti diagram showing global estimates of admixture proportions among African, European, and Native American ancestries in the CO/CR pedigrees. The global estimates were calculated using microarray data with ADMIXTURE software.

We verified the ethnicity of founders in our pedigrees using principal component analysis with 1KG^32^ (Supplementary Figure 8). We then estimated genome-wide ancestry proportions in members of these pedigrees using ADMIXTURE^37^. As expected^46^, the majority of ancestry was European, with a substantial Native American proportion and a small African proportion (Fig. 1). The admixture proportions in the pedigrees overall were associated with BP1 status; risk of BP1 increased by Odds ratio (OR) of 1.53 (GLMM *p* = 0.0008) with every increase of 0.1 units of European ancestry, whereas we observed the opposite trend for Native American ancestry with OR of 0.67 (GLMM *p* = 0.0096), and African ancestry with OR of 0.61 (GLMM *p* = 0.026) (Supplementary Figure 9).

### PRS analysis of BP1 and SCZ GWAS summary statistics

**Fig. 2:**
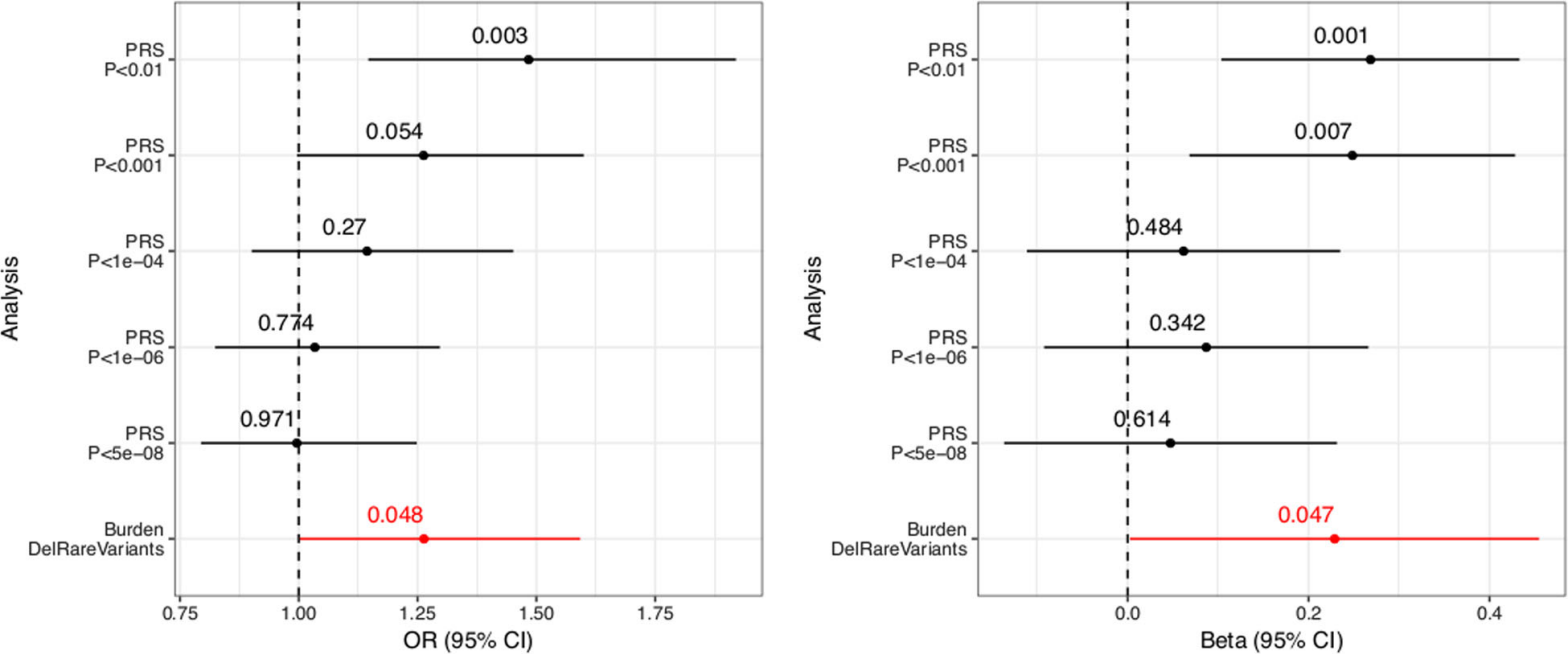
Forest plot displaying the mean and confidence interval of regression coefficients of PRS analysis and rare variant burden analysis for SNVs. We compared the quantile-normal transformed PRS estimated from PGC BP1 GWAS summary statistics between BP1 individuals and controls and also compared the burden of rare deleterious SNVs between BP1 individuals and controls in the 8,237 genes relevant to BP. PRS is computed at different GWAS *p* value thresholds of the PGC BP1 GWAS. The burden score was regressed on the burden of all rare variants in the 8,237 genes, and the residuals were quantile-normal transformed. The black lines indicate results of the PRS analysis while the red line indicates results of the rare variant burden analysis. The association between PRS and BP1 status and between the rare variant burden and BP1 status was assessed using a generalized linear mixed model (left) and a linear mixed model (right) that took into account relatedness.

To determine the effect of common SNPs on BP1 in the CO/CR pedigrees, we calculated PRS for each individual using the latest PGC GWAS summary statistics for BP1 (14,583 cases and 30,424 controls)^2^. We calculated PRS at different GWAS *p* value thresholds, where higher *p* value thresholds used more common variants in the PRS calculation. Results show that the mean PRS is higher in 190 BP1 individuals compared with 130 controls, at GWAS *p* value thresholds of 0.01 and 0.001 using LMM (*p* = 0.001 and 0.007, respectively) and at a GWAS *p* value threshold of 0.01 using GLMM (*p* = 0.003, Table 2, Fig. 2). We also calculated Nagelkerke’s *R*^2^ from logistic regression and found that these PRS explain 1.5% of the variance (Table 2). This *R*^2^ is noticeably smaller than that explained by PRS in the latest PGC BP GWAS data where the weighted average Nagelkerke’s *R*^2^ is 8%. Although this difference in the variance explained by the PRS could be owing to the population-level differences between the mostly European-descended PGC samples and the Latin America pedigrees in our study, >90% of SNPs in the PGC BP GWAS were present in our pedigrees (Table 2); it is therefore unlikely that this explanation, alone, explains the difference between the pedigree and population samples although we cannot ignore the possibility that different linkage equilibrium patterns or different frequencies of causal variants may contribute to this *R*^2^ difference.

**Table 2:** Comparison of Polygenic risk score estimated from PGC BP1 GWAS summary statistic between BP1 individuals and controls.

GWAS threshold	NSNPs, NSNPsPGC, NSNPsPGCInCOCR	LM beta (BP)	LM *P* value (BP)	LM Beta (AdMix)	LM *P* value (AdMix)	LR Log OR (QNPRS)	LR *P* value (QNPRS)	OR for 1-unit increase in QNPRS	LR Log or (AdMix)	LR *P* value (AdMix)	Nagelkerke *R*^2^
0.01	26868, 189221, 171166	0.27	0.0014	−4.11	2.13E-09	0.39	0.0027	1.477	5.53	5.12E-05	0.0151
0.001	4241, 36855, 34460	0.25	0.0069	−3.24	2.92E-05	0.23	0.0539	1.262	4.90	2.27E-04	0.0046
0.0001	789, 9457, 9017	0.06	0.4842	−2.83	1.23E-04	0.13	0.2704	1.143	4.54	4.52E-04	0.0007
0.000001	65, 896, 855	0.09	0.3421	−0.44	5.81E-01	0.03	0.7740	1.034	4.29	7.48E-04	0.0002
0.00000005	13, 126, 124	0.05	0.6136	−0.74	3.67E-01	0.00	0.9709	0.996	4.27	7.94E-04	0.0003

*P* values are computed using linear and logistic regression models by taking into account relatedness. Nagelkerke *R*^2^ calculation assumed independence among individuals. *NSNPs* the number of SNPs used in PRS calculation after LD clumping, *NSNPsPGC* the number of SNPs in the PGC data without LD clumping, *NSNPsPGCInCOCR* the number of SNPs from the PGC data present in CO/CR pedigrees without LD clumping, *BP* coefficients and *p* values for BP1 status, *AdMix* coefficients and *p* values for global admixture proportions of European ancestry, *LM* linear model, *LR* logistic regression, *QNPRS* quantile-normalized polygenic risk scores.

Although evidence over several decades delineated the distinctions between BP and SCZ, more recent studies have highlighted genetic overlaps between these syndromes^2,47,48^, which share symptoms in common. Notably, in GWAS data from large BP case/control samples, the PRS estimated from the PGC’s SCZ GWAS results have explained up to 2.5% of BP variance^47^. We contrasted the mean PRS from SCZ GWAS in BP1 individuals and related controls in our pedigrees; to calculate PRS we used the GWAS summary statistics for SCZ^44^ (36,989 cases and 113,075 controls). These SCZ PRS are not statistically associated with an increased risk of BP1, in the CO/CR pedigrees, at any of the GWAS *p* value thresholds that we examined (Supplementary Table 8). The association of SCZ PRS with BP1 in PGC samples, but not in the CO/CR pedigrees may suggest differences in the characteristics of BP1 between these samples; in particular, this contrast between our results and those of the PGC may reflect the fact that we ascertained each of the pedigrees for multiple closely related cases of BP1.

### Burden of rare deleterious SNVs and rare CNVs in the gene-set for BP1

We first identified 8,757 genes related to BP1 using the three sources of information based on the CNS cell type region where BP1 heritability is enriched, regions near PGC BP1 GWAS signals, and regions near linkage peaks from these pedigrees (see Methods). We then identified rare SNVs in those genes using both an external source of allele frequency and allele frequency observed in the CO/CR families (see Methods). We identified 25,072 rare predicted-deleterious SNVs in 8,237 of the 8,757 genes in our gene-set. For each individual, we computed the mean genome-wide burden of these SNVs, then compared these means between BP1 individuals and related controls, whereas taking into account the proportion of European ancestry in each individual, and the mean genome-wide burden of all rare SNVs in the gene-set. We observed that the mean burden of the rare deleterious SNVs was higher in BP1 individuals than in controls (*p* = 0.047 using LMM, Fig. 2). The risk of BP1, as indicated by OR increased by 1.26 for every one unit increase in quantile-normal transformed residual mean burden (*p* = 0.048 using GLMM). We also tested the burden of rare deleterious SNVs in genes defined by each of the three sources for the gene-set and observed a higher burden only in genes from the CNS cell type region (*p* = 0.040), but not in genes near PGC BP1 GWAS associations (*p* = 0.648) or in genes near linkage peaks (*p* = 0.399). This is expected as a majority of genes in our gene-set came from the CNS cell type region.

**Fig. 3:**
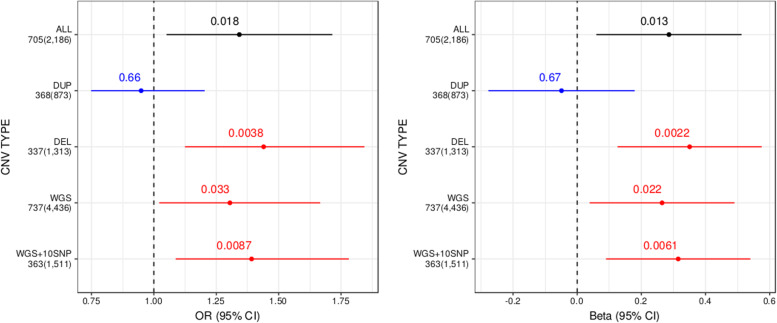
Forest plot displaying the mean and confidence interval of regression coefficients of rare variant burden analysis for CNVs. We compared the burden of genes affected by rare CNVs in the BP1-related gene set between BP1 individuals and controls, stratified by a detection method (microarray or WGS) and a CNV type. The total number of CNVs detected and the number of CNVs affecting BP1-related genes are displayed for each category. The black line indicates results of all microarray CNVs, the blue line indicates results of duplications from microarray, red lines indicate results of deletions from microarray (DEL) and WGS (WGS and WGS + 10SNP) data. WGS + 10SNP is results of WGS deletions covered by at least 10 SNPS on the microarray. To correct for individual relatedness and other potential confounders (see Methods), enrichment was assessed using a generalized linear mixed model (left) and a linear mixed model (right).

We also performed an analysis of genome-wide burden using rare CNVs detected from microarray and WGS. Our CNV burden score was calculated as the number of genes in our BP1 gene-set intersected by rare CNVs, and similar to the burden analysis of rare deleterious SNVs, we measured the enrichment of CNV burden score using LMM and GLMM, accounting for factors known to affect global measures of CNV burden (see Methods). For CNVs from microarray (2,186 rare CNVs among 189 BP1 individuals and 128 controls), BP1 individuals had a higher CNV burden score than controls (LMM *p* = 0.013 and GLMM *p* = 0.018 with OR of 1.34, Fig. 3, Supplementary Table 9). Stratifying our analysis by CNV type, we observed that this increased burden was attributable exclusively to deletions (LMM *p* = 2.2e-3 and GLMM *p* = 3.8e-3 with OR of 1.44). For CNVs from WGS (4,436 rare deletions among 190 BP1 individuals and 130 controls), we also observed an increased burden of genes in the BP1 gene-set affected by rare CNVs for BP1 individuals (LMM *p* = 0.022 and GLMM *p* = 0.033 with OR of 1.3, Fig. 3). This burden was greater (LMM *p* = 6.1e-3 and GLMM *p* = 8.7e-3 with OR of 1.39) when restricting our analysis to the subset of CNVs covered by a minimum of 10 SNPs on microarray (*n* = 1,511), thus demonstrating a consistent increase in gene count burden using different methods of detection. We did not, however, observe a difference in the average number of all rare CNVs between BP1 individuals and controls (*p* = 0.67 for microarray CNVs and *p* = 0.45 for WGS CNVs using GLMM).

### Segregation of rare deleterious SNVs and CNVs in the gene-set for BP1

To detect the segregation of rare variants with BP1 in the CO/CR pedigrees, we developed a statistical approach that quantifies the significance of the observed segregation pattern. Intuitively, it estimates the probability that we would observe the given segregation pattern of a rare variant or more extreme patterns under the null hypothesis of random segregation, we refer to this as the segregation *p* value (see Methods and Supplementary Text). Among the rare SNVs and CNVs analyzed in the previous burden analysis, we filtered out variants not shared between BP1 individuals (Supplementary Table 10) and also variants for which we were not able to identify founders who introduced rare variants into the family with high confidence (Supplementary Figures 10 and 11). In total, we analyzed segregation for 6,421 rare deleterious SNVs in 4,050 genes and 314 rare CNVs in 251 genes.

No segregation *p* value for either SNVs or CNVs passed the significance threshold after the Bonferroni correction (Supplementary Tables 11 and 12). The top gene in the SNV segregation analysis was *ACTR1B* (*p* = 5.18e–04), which contained one rare missense variant (rs141238033, chr2:98275876). This variant did not appear in the Colombian samples within 1000 Genomes and was very rare in the Latino samples of ExAC (MAF of 0.04%). It was enriched in the CO/CR pedigrees as MAF in all 449 sequenced individuals was 0.44% accounting for relatedness^49^ (11× over ExAC) and 1.89%, not accounting for relatedness (47× over ExAC). *GOLPH3* was the top gene in the CNV segregation results (*p* = 7.89e-4), with a single rare CNV (DEL_P0095_217, chr5:32161816-32162478) appearing only in family CO27 (MAF of 0.22% accounting for relatedness and 0.78% not accounting for relatedness). Neither of the above two rare variants segregated perfectly with BP1 status in the pedigrees in which they were present (Supplementary Tables 13 and 14).

## Discussion

We demonstrate that common variants in extended pedigrees contribute to BP1 risk while observing modest evidence of effect of rare variants on BP1 risk. Elevated BP1 PRS scores, in BP1 individuals compared with controls, indicate that as in case/control samples, some of BP1 risk derives from the polygenic effect of common SNPs, with Nagelkerke’s *R*^2^ of 1.5%. This result is in accordance with observations in pedigree studies of non-psychiatric common disorders^50^. It remains unclear, however, why the magnitude of the polygenic contribution is so much smaller in our pedigrees compared with cases from the PGC. The dissimilarity between these study samples in size and ethnicity could explain this divergence^51^, as could the close relationship between our BP1 cases and their control relatives, who might carry some polygenic burden of BP1. At last, it is possible that BP1 in individuals from extended pedigrees is simply less polygenic than in population samples.

In support of the idea that BP1 in pedigrees may be etiologically distinct from BP1 in population samples, we did not observe effect of common SCZ risk variants on BP1 risk in our samples. This result contrasts with the PGC finding that the SCZ PRS is a strong predictor of BP1 risk as the BP PRS. This discrepancy relates to an important uncertainty regarding severe mental illness, the genetic relationship between BP and SCZ. The separation between BP and SCZ has been a bedrock principle of psychiatric nosology, based on the distinct trajectories of these syndromes and genetic epidemiology studies suggesting that they do not co-segregate in families. Recent studies, however, indicate a shared genetic architecture between SCZ and BP1^2,47,48^. Efforts now underway in multiple datasets are examining the relationship between BP and SCZ at a finer-grained level than that of syndromic diagnosis^52^.

No convincing rare BP susceptibility variants, or even loci, have yet been reported from either pedigree or case/control sequencing studies. However, the comprehensive genotype data that our study contains provide an opportunity for more complete evaluation than has previously been possible of the contribution of rare variants to BP1 within pedigrees, and for the segregation of rare variants with the disorder.

In assessing the contribution of rare variants to BP1, we found, in a set of 8,757 genes selected based on hypothesized relevance to this disorder, some evidence of collective effect of rare variants (deleterious SNVs as well as CNVs) by comparing the burden of those variants carried by affected individuals and that by related controls. We chose this set of genes because they corresponded to regions where BP1 heritability was enriched and where BP1 GWAS hits and our linkage peaks resided. This approach assumes that genes affected by rare variants overlap with those affected by common variants, which was observed in other studies such as human height^53^ and SCZ^54^. Ament et al.^55^, in a gene-set related to neuronal excitability, observed a similar enrichment of rare BP risk variants. Previous studies reported no evidence for a global enrichment of rare CNVs in BP individuals, but analyzed only CNVs > 100 kb^56,57^. Our results suggest that the impact of CNVs on BP burden derives mainly from CNVs of 5–100 kb, and may be restricted to specific gene-sets.

We attempted to discover the specific loci and variants responsible for increased rare variant burden for BP1 in our data set. We used a new statistical approach that we developed to calculate *p* values for rare variant segregation, because existing methods^58–60^ are not scalable to our large pedigrees and also make the simplifying assumption that only one founder has introduced a given rare variant into the pedigree. Our method relies on accurate imputation of rare variants, achieved using a family imputation approach that achieves a higher call rate and accuracy for rare alleles than population-based imputation approaches^23^. Our method worked well, from a technical standpoint but did not detect rare variants with strong evidence of segregation in our pedigrees after correction for multiple testing. One reason may be that there are 320 individuals who could be designated as either BP1 or controls, in the total sample of individuals (782) who are either sequenced or imputed well. If phenotypic status was more definite in a higher proportion of these individuals it could have added substantially to the power to detect such associations.

In conclusion, results of our study point to the polygenic genetic architecture of BP1 in a well-characterized and large series of extended pedigrees, reflecting the action of a combination of many common and possibly rare variants (including both SNVs and CNVs) with small or moderate effect sizes. Rare variants with relatively large effect may contribute substantially to genetic risk of BP1 in the pedigrees although identifying associations to those rare variants is likely to require larger samples than were available in the current study. Identifying these variants may also require advances in our ability to recognize functionally important variation in non-coding parts of the genome. In addition, unlike in BP case/control samples, common SCZ risk alleles appear to contribute less than the weak effect of common variants we observed for BP1 in these families. Finally, although our new method makes it feasible to rigorously evaluate rare variant segregation in large pedigrees, our inability to identify BP1-associated coding variants suggests that non-coding variants may play an important role in BP1 risk in these pedigrees.

## Supplementary information

Supplemental Information

Supplementary Table 6
