# Supplementary material for: Contribution of common and rare variants to bipolar disorder susceptibility in extended pedigrees from population isolates
Source: Transl Psychiatry. 2020 Feb 24;10:74. doi: 10.1038/s41398-020-0758-1 (PMC7039961; doi:10.1038/s41398-020-0758-1)
Supplement: Supplementary file 1 — Supplemental Information [file 41398_2020_758_MOESM1_ESM.pdf]

SUPPLEMENTAL INFORMATION

Supplementary Figures

Supplementary Figure 1. An overview of steps to produce final QC’d array genotype and WGS data sets.

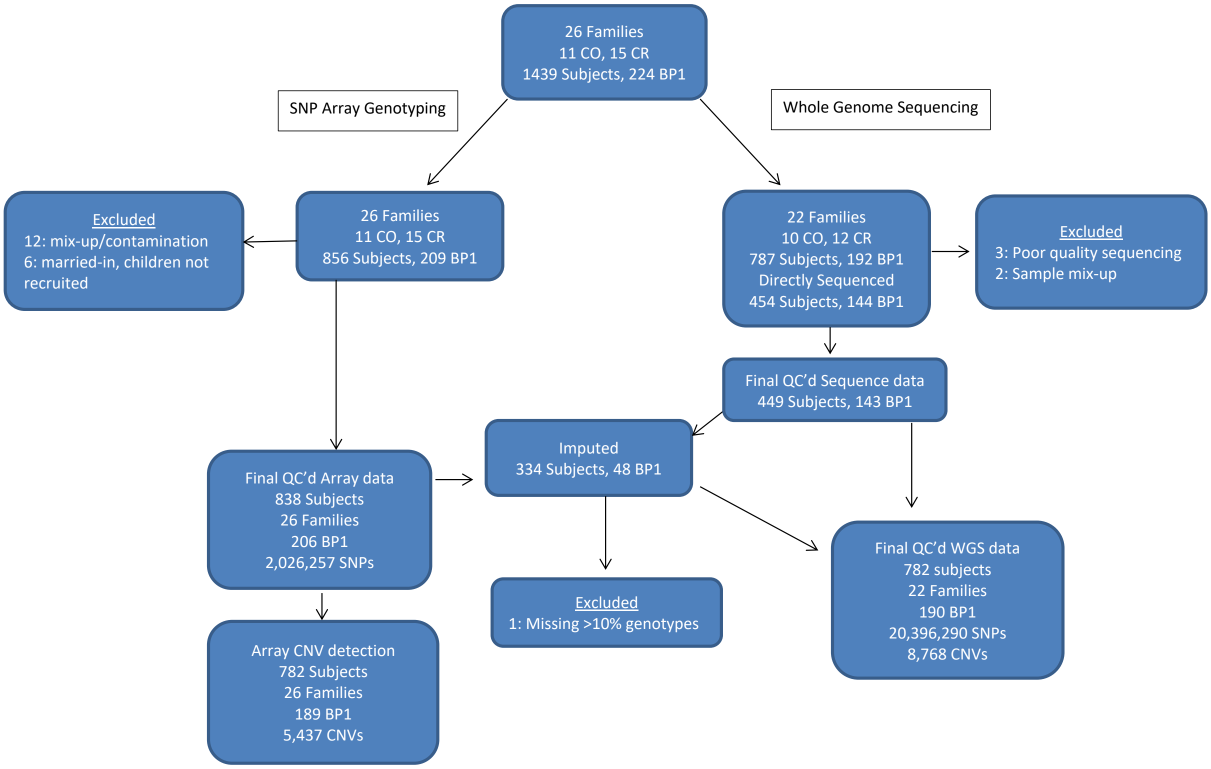

Supplementary Figure 2. Comparison of genotype missing rate of individuals from imputed data and the genotype concordance rate between genotypes imputed by GIGI and microarray genotypes. Each point represents each imputed individual. Genotypes were missing if genotype probability from GIGI imputation was lower than the genotype probability threshold, and we included only individuals whose genotype missing rates were < 10% for subsequent analyses.

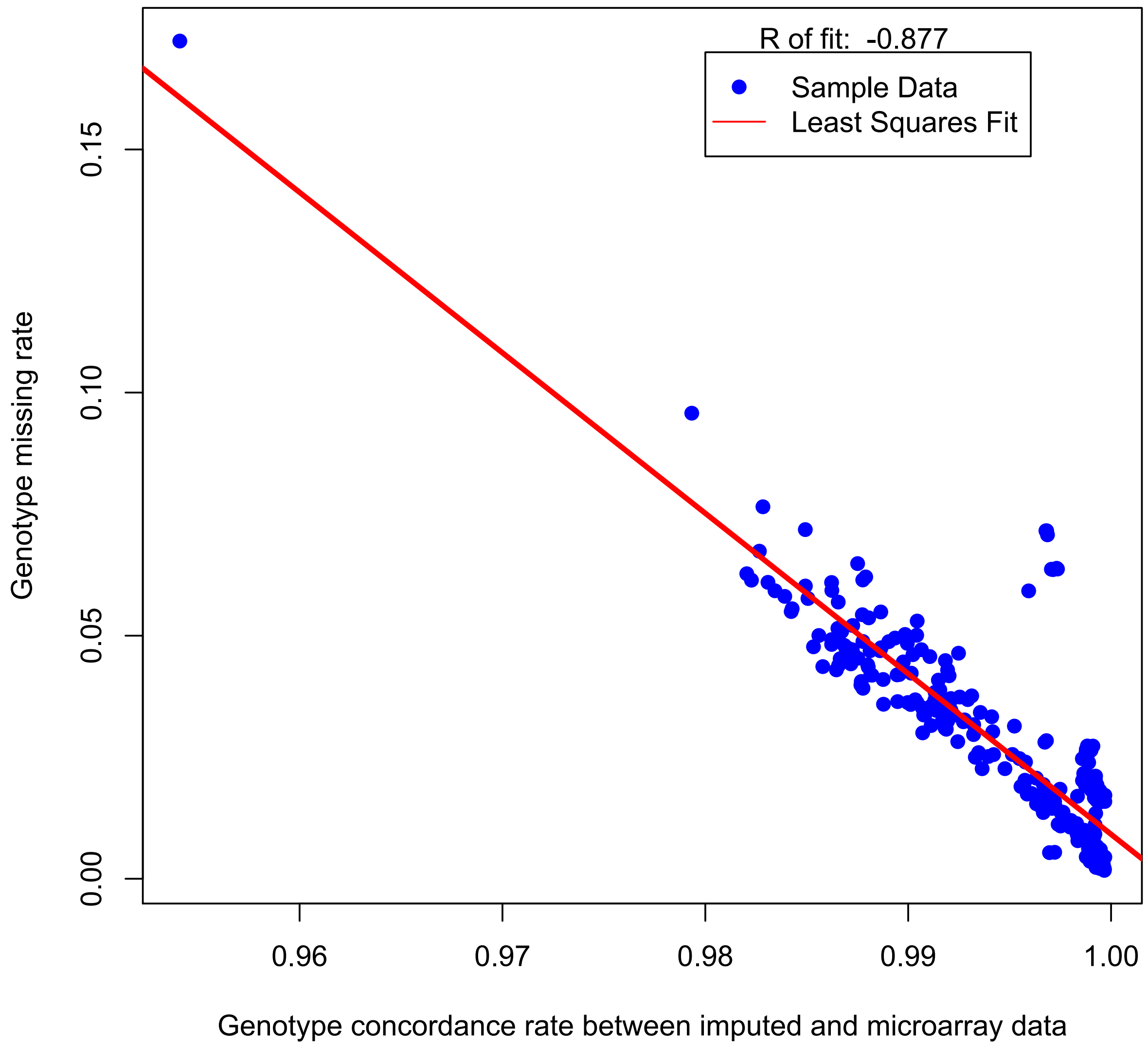

Supplementary Figure 3. Array-based CNV detection sample QC. We excluded outlying samples based on manual inspection of the distributions across all samples on three different intensity-based QC metrics: (A) Log-R ratio standard deviation (LRR_SD), a general measure of intensity signal to noise; (B) B-allele frequency standard deviation (BAF_SD), the deviation of heterozygous B-allele frequency measures from the expect value of 0.5; (C) waviness (bottom), which measures the waviness of signal intensities after accounting for local GC-bias. (D) Additionally, we removed samples with an excessive number of rare CNV calls.

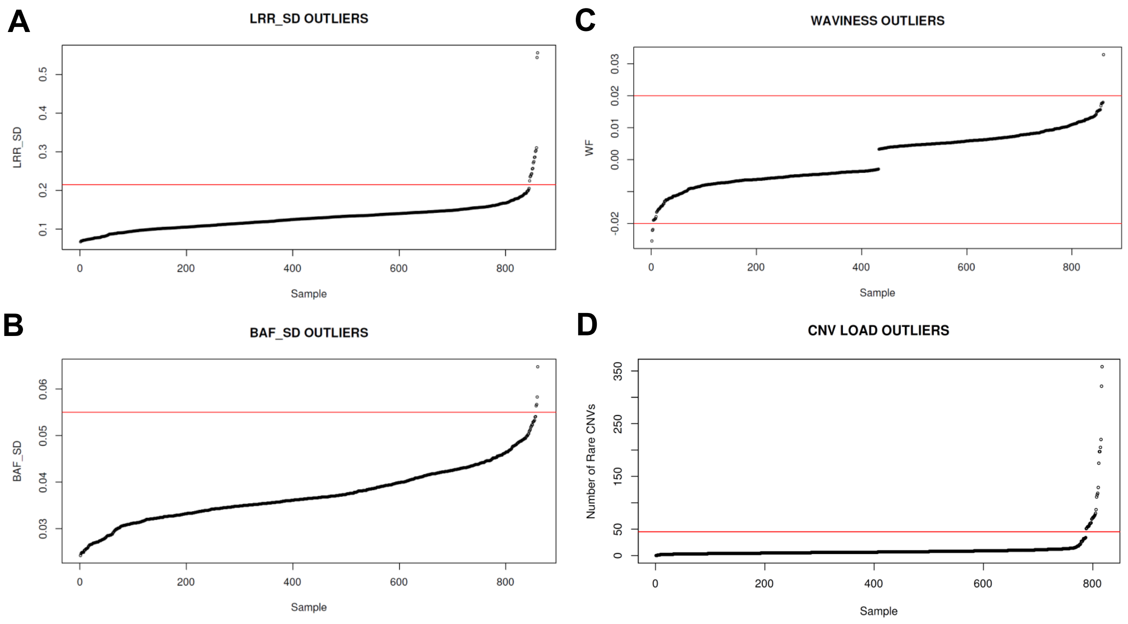

Supplementary Figure 4. WGS CNV detection sample QC. (A) Following genome-wide detection of deletion CNVs from WGS, we removed five samples with an excessive number of CNV calls or with possible sample mix-up who also all failed SNV QC prior to imputation in additional samples with available genotype data (see Methods). (B) Per chromosome plot of total number of CNV calls detected by WGS across remaining samples reveals no substantial sample outliers. Peaks on chromosome 2 and chromosome 14 correspond to VDJ recombination regions. CNV calls within these regions were excluded from further analysis. (C) Rate of Mendelian-inconsistent CNV genotype calls across 67 complete trios available from all 449 sequenced individuals. The average rate of inconsistent sites across all genotyped sites was 0.0026±0.00067.

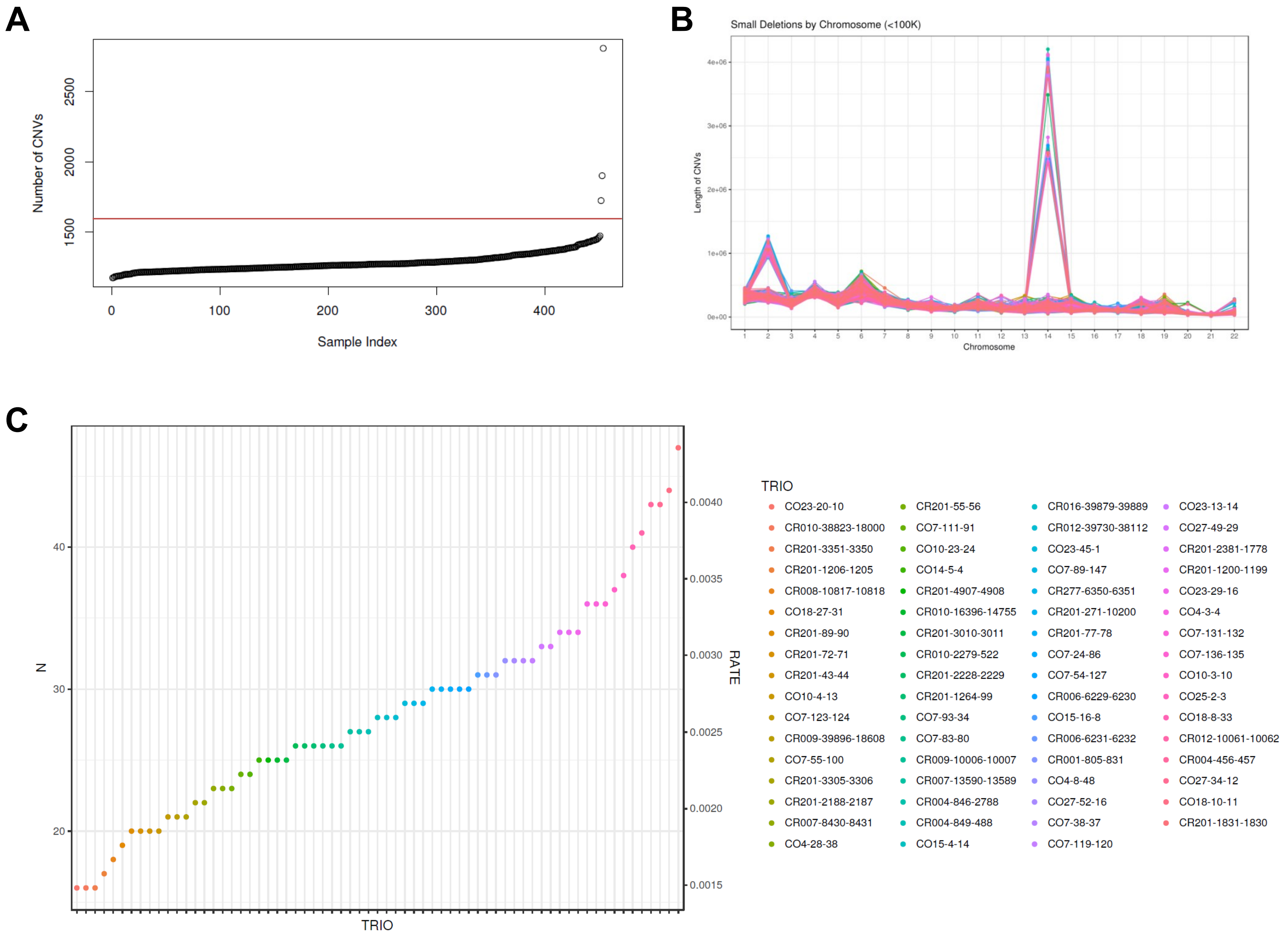
A
Number of CNVs
Sample Index
B
Small Deletions by Chromosome (<100K)
Length of CNVs
Chromosome
C
N
RATE
TRIO
CO23-20-10
CR010-38823-18000
CR201-3351-3350
CR201-1206-1205
CR008-10817-10818
CO18-27-31
CR201-89-90
CR201-72-71
CR201-43-44
CO10-4-13
CO7-123-124
CR009-39896-18608
CO7-55-100
CR201-3305-3306
CR201-2188-2187
CR007-8430-8431
CO4-28-38
CR201-55-56
CO7-111-91
CO10-23-24
CO14-5-4
CR201-4907-4908
CR010-16396-14755
CR201-3010-3011
CR010-2279-522
CR201-2228-2229
CR201-1264-99
CO7-93-34
CO7-83-80
CR009-10006-10007
CR007-13590-13589
CR004-846-2788
CR004-849-488
CO15-4-14
CR016-39879-39889
CR012-39730-38112
CO23-45-1
CO7-89-147
CR277-6350-6351
CR201-271-10200
CR201-77-78
CO7-24-86
CO7-54-127
CR006-6229-6230
CO15-16-8
CR006-6231-6232
CR001-805-831
CO4-8-48
CO27-52-16
CO7-38-37
CO7-119-120
CO23-13-14
CO27-49-29
CR201-2381-1778
CR201-1200-1199
CO23-29-16
CO4-3-4
CO7-131-132
CO7-136-135
CO10-3-10
CO25-2-3
CO18-8-33
CR012-10061-10062
CR004-456-457
CO27-34-12
CO18-10-11
CR201-1831-1830

Supplementary Figure 5. Enrichment z-scores of heritability of BP in 10 different cell type groups using the stratified LD score regression. The horizontal dotted line is the threshold for significant enrichment. CNS: central nervous system. GI: Gastrointestinal.

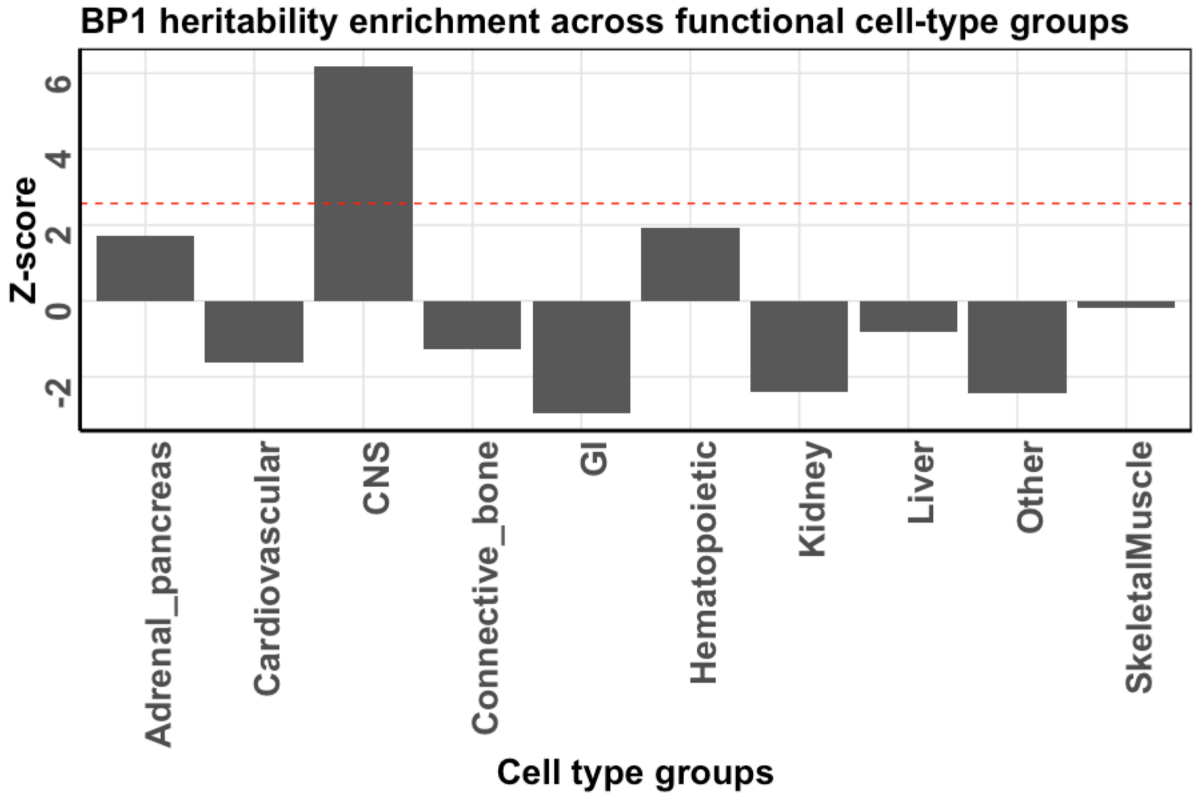

Supplementary Figure 6. Manhattan plot of genome-wide two-point parametric heterogeneity linkage analysis with 99,446 SNVs (circles) and 85,813 STRs (triangles). The horizontal line at HLOD=4.1 represents genome-wide significance.

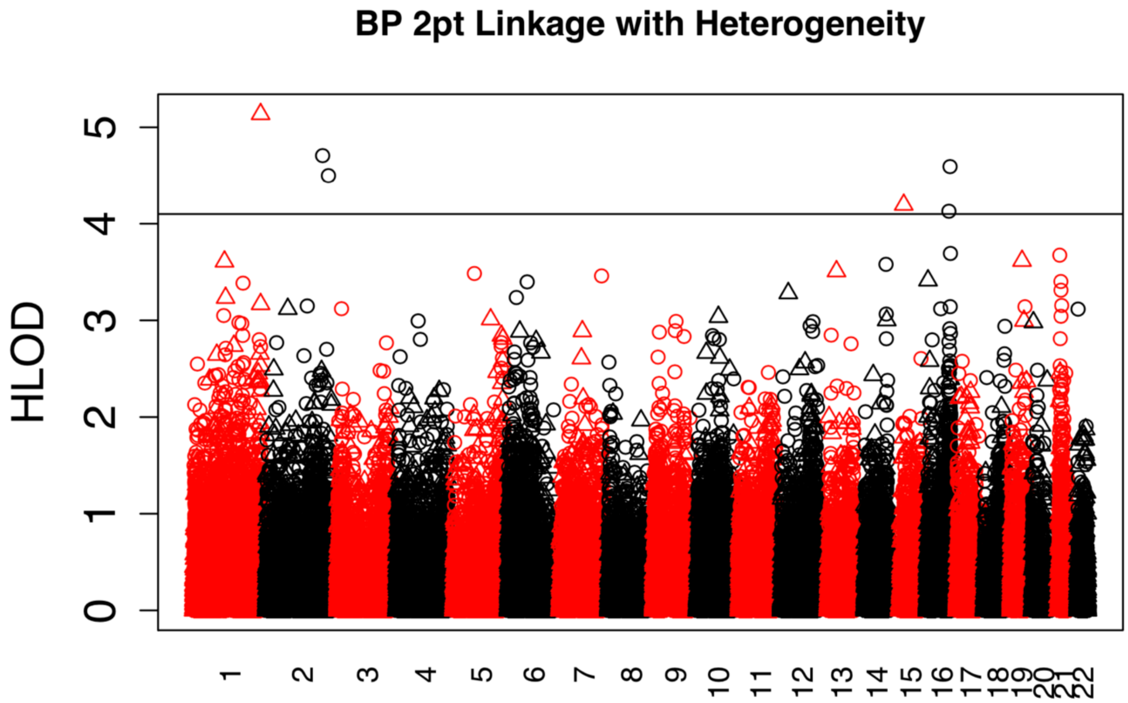

Supplementary Figure 7. An example of a pedigree for the segregation analysis. In this family, among 10 individuals, only the latest offspring (individuals 9 and 10) are affected while all others have a missing phenotype, which generates the maximum S_{rare} statistic of 2. The p-value of S_{rare} statistic of 2 is much more significant when the top founder (founder 1) is F^{rv} (founders with a rare variant) compared to when a parent of individuals 9 and 10 (founder 8) is F^{rv}. The fact that the rare variant is inherited in every generation and shared among the two affected individuals in the last generation is a more rare event than when the rare variant is directly inherited from a parent and hence yields a more significant p-value.

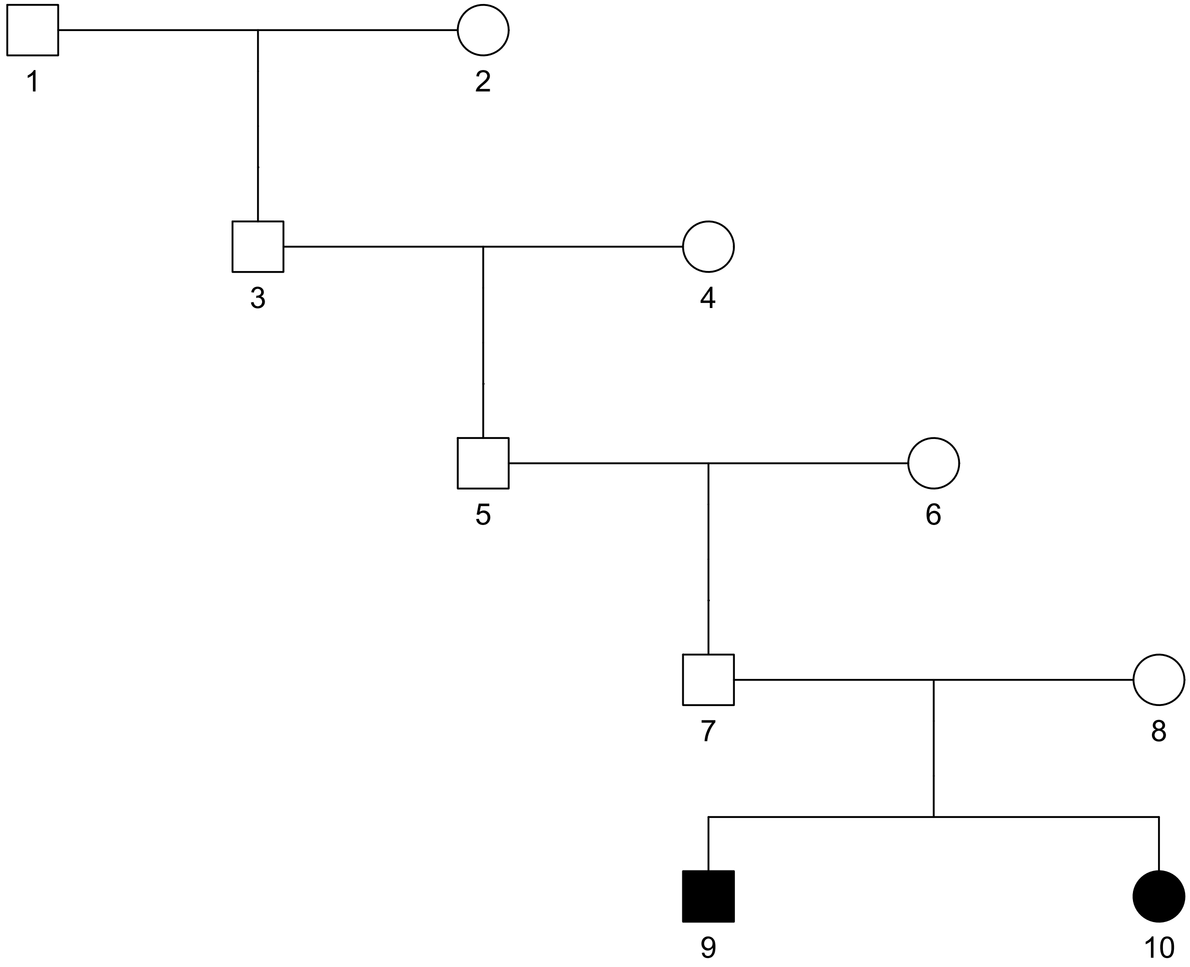

Supplementary Figure 8. First two dimensions of a PCA analysis of founders from the BP1 WGS data set, using 1000 Genomes phase 3 samples as reference.

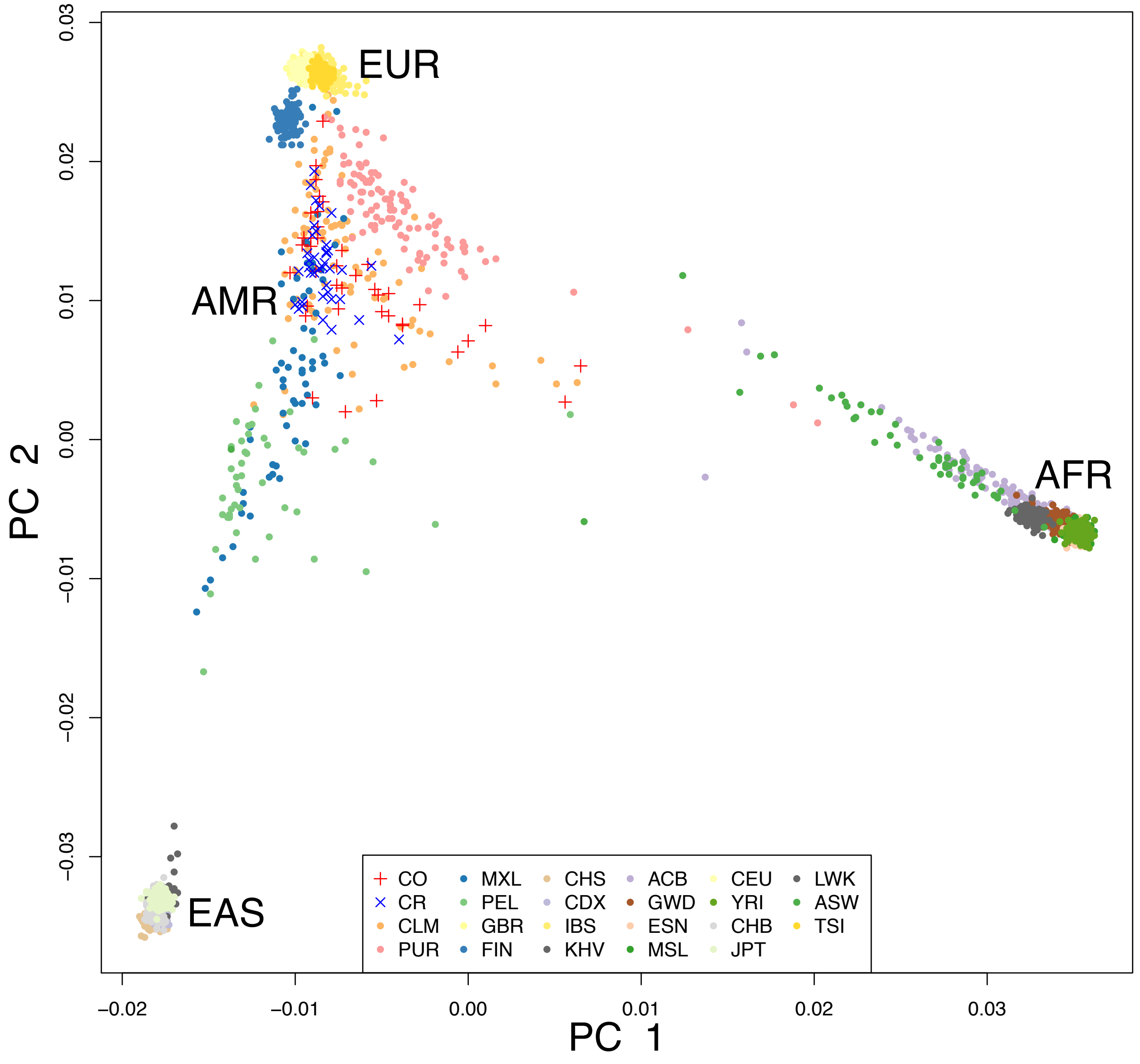

Supplementary Figure 9. Comparison of global estimates of admixture proportions between BP1 individuals and controls. Admixture proportions were compared for African, European, and Native American ancestries separately.

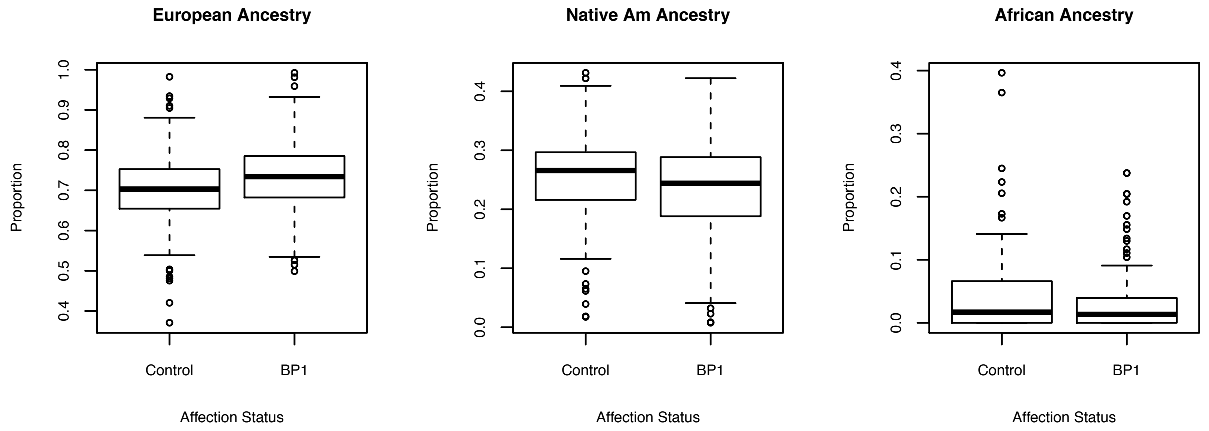

Supplementary Figure 10. The number of rare SNVs passing the three different filters. The first filter (F1) is the number of rare variants with genotype missing rate < 5% and in which at least two affected individuals in a family share the variant. The second filter (F2) is the number of rare variants for which all founders have high-quality genotypes (the highest genotype probability among the three probabilities from GIGI imputation > 0.8). The third filter (F3) is the number of rare variants for which all founders except a pair of top founders have high-quality genotypes. The “Passing F1 & (F2 or F3)” filter is the number of rare variants that we analyzed in the segregation analysis.

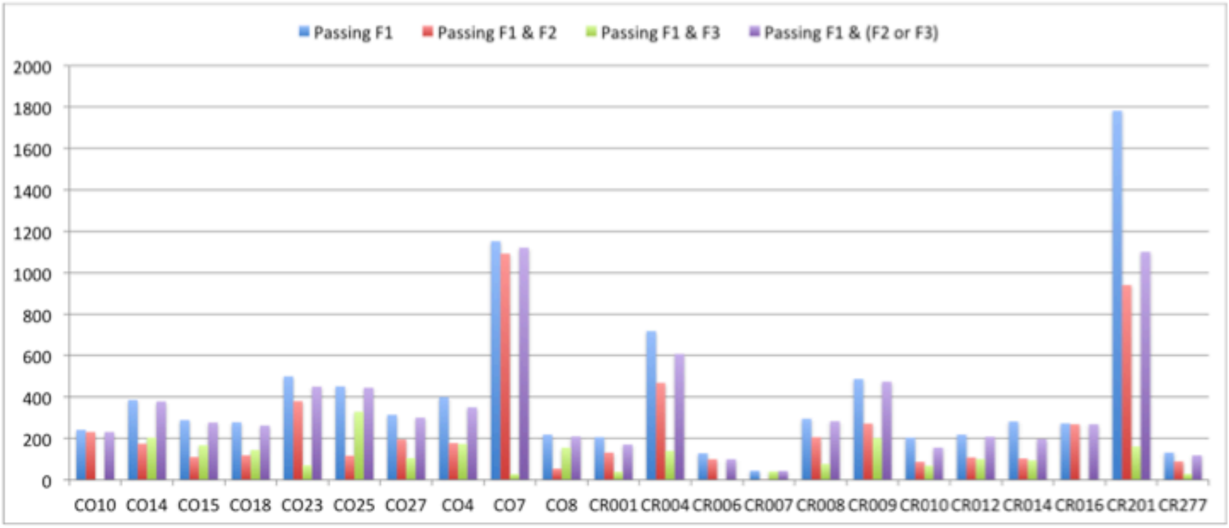

Supplementary Figure 11. The number of rare CNVs passing the three different filters. The first filter (F1) is the number of rare variants with genotype missing rate < 5% and in which at least two affected individuals in a family share the variant. The second filter (F2) is the number of rare variants for which all founders have high-quality genotypes (the highest genotype probability among the three probabilities from GIGI imputation > 0.8). The third filter (F3) is the number of rare variants for which all founders except a pair of top founders have high-quality genotypes. The "Passing F1 & (F2 or F3)" filter is the number of rare variants that we analyzed in the segregation analysis.

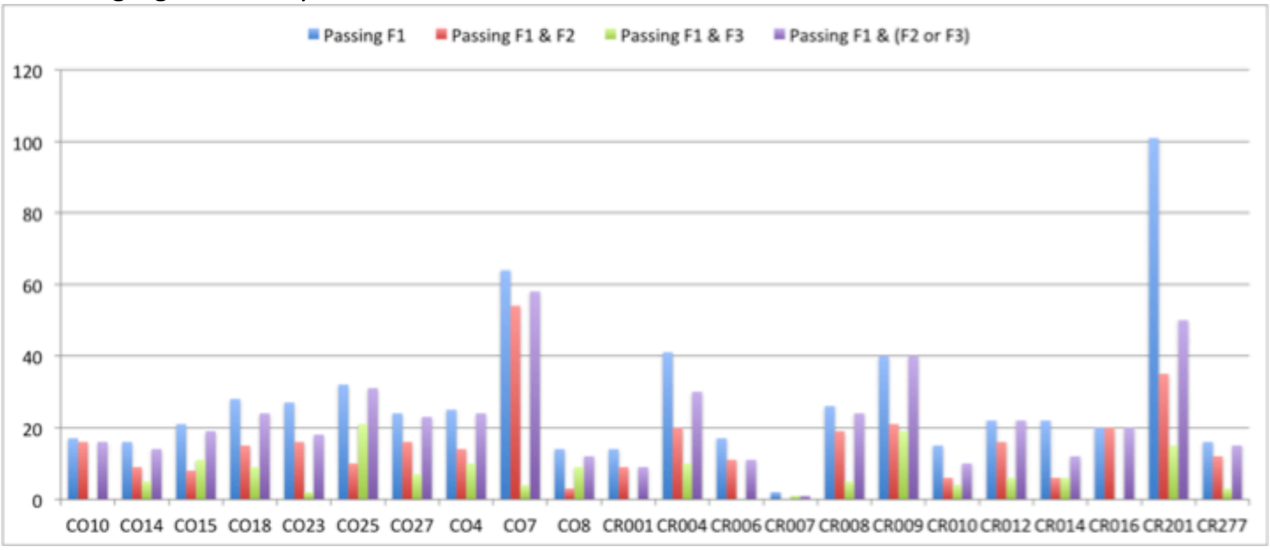

Supplementary Tables

Supplementary Table 1. Summary QC metrics on SNVs to pass all QC. Mean values are taken over 449 individuals with sequence data after removing five individuals with poor sequencing quality and sample mix-up, and all variant sites that passed QC. NVariants: Number of Variant Sites; MeanDepth: Mean Depth; MeanGQ: Mean Genotype Quality; NArrayVariants: Number of Variant Sites seen on array chip; Pct01Discordant: Percent of markers with 0 or 1 discordant genotypes compared to array data; PctMissingMT01: Percent of markers missing more than 1% of data; Pct01ME: Percent of markers with 0 or 1 Mendelian transmission errors in 67 trios; PctHWELT104: Percent of markers with HWE p-value <0.0001.

CHR	NVariants	MeanDepth	MeanGQ	NArrayVariants	Pct01Discordant	PctMissingMT01	Pct01ME	PctHWE
1	1,597,894	36.49	93.19	142,772	99.45%	1.57%	99.89%	0.01%
2	1,747,882	36.76	93.70	152,091	99.45%	1.42%	99.88%	0.01%
3	1,490,005	36.89	93.92	128,855	99.45%	1.30%	99.84%	0.01%
4	1,476,620	37.07	94.12	119,880	99.44%	1.29%	99.89%	0.01%
5	1,327,700	36.85	93.91	113,159	99.44%	1.29%	99.93%	0.00%
6	1,294,148	36.94	93.87	113,983	99.36%	1.57%	99.83%	0.02%
7	1,196,061	36.64	93.33	101,483	99.43%	1.71%	99.84%	0.01%
8	1,167,413	36.79	93.72	99,111	99.40%	1.30%	99.93%	0.02%
9	865,972	36.55	93.32	80,725	99.40%	1.51%	99.89%	0.00%
10	1,026,662	36.59	93.31	94,758	99.44%	1.52%	99.91%	0.01%
11	994,937	36.62	93.38	89,614	99.48%	1.54%	99.87%	0.01%
12	971,757	36.66	93.45	87,546	99.43%	1.65%	99.90%	0.01%
13	735,365	37.00	94.01	65,846	99.38%	1.34%	99.89%	0.00%
14	641,845	36.71	93.38	58,817	99.46%	1.90%	99.88%	0.00%
15	584,891	36.61	93.21	56,091	99.38%	1.60%	99.79%	0.00%
16	654,306	36.20	92.26	59,621	99.33%	1.73%	99.89%	0.05%
17	566,990	35.61	91.65	51,786	99.25%	1.92%	99.89%	0.02%
18	577,069	36.82	93.79	54,736	99.41%	1.39%	99.90%	0.01%
19	470,547	35.00	90.28	36,853	99.19%	2.90%	99.54%	0.01%
20	460,660	36.07	92.61	45,241	99.42%	1.65%	99.90%	0.01%
21	280,646	36.81	93.36	25,354	99.47%	1.58%	99.89%	0.01%
22	266,920	35.16	90.97	26,531	99.27%	2.37%	99.83%	0.02%
X	510,990	29.61	77.52	34,376	99.99%	0.02%	100.00%	0.00%

Supplementary Table 2. Average GQ and DP of genotypes that were changed and unchanged by Polymutt. Mode 0 is a case where Polymutt did not change the original genotypes. Mode 1 is a case where Polymutt changed genotypes. Mode 2 is a case where Polymutt imputed genotypes. A genotype is Mendelian inconsistent if it belongs to a trio or a pair with an Mendelian error on the variant. It is Mendelian consistent otherwise.

Mode	Original Genotype	After Polymutt	Average GQ of Mendelian consistent genotypes	Average GQ of Mendelian inconsistent genotypes	Average DP of Mendelian consistent genotypes	Average DP of Mendelian inconsistent genotypes
0	A/A	A/A	93.06	81.14	36.47	32.6
1	A/A	A/C	51.89	53.65	18.61	25.02
2	Missing	A/A	8.97	6.13	28.81	25.33

Supplementary Table 3. False Discovery Rate (FDR) of CNV detection by WGS. CNV_LOCI: The total number of CNV loci considered for FDR estimation; IRS_FDR: The genome-wide estimate of FDR, calculated using an intensity rank-sum test on available array data.

MINIMUM_SIZE (KB)	CNV_LOCI	IRS_FDR
0	8768	0.0397
1	6480	0.0270
2	4311	0.0207
3	3198	0.0181
4	2484	0.0185
5	2010	0.0179
6	1618	0.0175
7	1297	0.0069
8	1117	0.0080
9	975	0.0068
10	832	0.0053
15	526	0.0043
20	399	0.0000
MINIMUM_PROBES	CNV_LOCI	IRS_FDR
1	4949	0.0397
2	3432	0.0097
3	2491	0.0067
4	1888	0.0077
5	1514	0.0083
6	1209	0.0104
7	1002	0.0063
8	853	0.0074
9	741	0.0085
10	649	0.0097

Supplementary Table 4. A summary on different types of genetic variants analyzed in this study. The Polymutt row indicates whether Polymutt was applied to a type of variant, and the Imputed row indicates whether GIGI imputation was applied to a type of variant. # of samples, BP1 individuals, controls, and variants is after QC. PRS: polygenic risk scores, RV: rare variant.

	WGS SNV	Array SNP	WGS CNV	Array CNV	STR
Software	GATK	GenomeStudio	GenomSTRiP	PennCNV, QuantiSNP	lobSTR
Polymutt	Yes	No	No	No	No
Imputed	Yes	No	Yes	No	No
# of samples	782	838	782	782	449
# of BP1 subjects	190	206	190	189	143
# of controls	130	NA	130	128	NA
Type of allele	Bi-allele	Bi-allele	Bi-allele (deletion)	Deletion and duplication	Bi-allele and multi-allele
# of variants	20,396,290	2026257 (99,446 for linkage)	8,768	5,437	86,601
QC	VQSR + classifier	Pagani et al. PNAS 2016	CNV QC	Huang et al. Neuron 2017	Comparison to electrophoresis STR calls
Analysis	PRS, RV burden and segregation	Linkage	RV burden and segregation	RV burden	Linkage

Supplementary Table 5. Results of parametric linkage and non-parametric linkage (NPL) analyses. For parametric linkage, SNVs and STRs with heterogeneity LOD score (HLOD) exceeding 4.1 are shown, and for NPL, markers with empiric p-values < 4.9e-05 are shown. Empiric p-values were initially tested with 250,000 simulations and also with 1,000,000 simulations. To check overlap between parametric linkage and NPL analyses and also between SNVs and STRs, we looked 1Mb to either side of each linkage peak to obtain the maximal evidence for linkage from other types of analysis or genetic variant. All BP positions are on build 37, hg19.

Parametric Linkage – SNV						
rsID	CHR	BP	HLOD	Alpha	Min NPL p-value	Max STR HLOD
kgp5681631	2	206823264	4.71	1	0.00036	2.04
kgp10402924	2	228285348	4.5	1		
kgp2684721	16	79556425	4.13	0.88	0.00032	2.39
rs8058020	16	83739599	4.59	0.92		
Parametric Linkage – STR						
rsID	CHR	BP	HLOD	Alpha	Min NPL p-value	Max SNV HLOD
STR	1	247103258	5.03	0.72	0.0024	2.73
STR	15	35265465	4.32	0.62	0.03327	0.71
NPL – SNV						
rsID	CHR	BP	emp_pvalue 250,000 simulations	emp_pvalue 1,000,000 simulations	Max STR HLOD	Max SNV HLOD
rs804129	1	15183309	2.80E-05	2.80E-05	0.54	1.86
rs3820546	1	43398085	3.20E-05	3.90E-05	1.13	1.97
kgp10316684	1	182297222	8.00E-06	1.10E-05	1.16	3.39
rs10056131	5	28302858	1.20E-05	2.20E-05	0.63	0.64
kgp9442190	6	31055539	2.80E-05	3.10E-05	1.16	2,42
rs12444317	16	77705268	0	2.00E-06	0.84	2.5
kgp3613333	21	23959348	8.00E-06	2.00E-06	1.39	3.15
kgp10714796	21	24623957	1.60E-05	1.80E-05		
NPL – STR						
rsID	CHR	BP	emp_pvalue 250,000 simulations	emp_pvalue 1,000,000 simulations	Max STR HLOD	Max SNV HLOD
STR	18	72256481	4.00E-05	2.60E-05	0.99	2.59

Supplementary Table 7. A summary on types of variants (common or rare), the number of tests, and the type of multiple testing correction applied to each analysis in this study. LMM: linear mixed models.

Analysis	Question of interest	Type of variants	The number of tests	Type of multiple testing correction
Admixture			3	None
PRS - BP1 summary statistic	Genetic architecture	Common	5	None
PRS - SCZ summary statistic	Genetic architecture	Common	5	None
Partitioned heritability using LD score regression			10	Bonferroni
Rare variant burden - SNV	Genetic architecture	Rare	4	None
Rare variant burden - CNVs in gene set	Genetic architecture	Rare	5	None
Rare variant burden - overall # of CNVs	Genetic architecture	Rare	2	None
Rare variant segregation - SNVs	Specific locus	Rare	6,421	Bonferroni
Rare variant segregation - CNVs	Specific locus	Rare	314	Bonferroni
Linkage analysis - parametric	Specific locus	Common	99,446 SNPs and 85,813 STRs	Lander-Kruglyak 1995
Linkage analysis - nonparametric	Specific locus	Common	99,446 SNPs and 85,813 STRs	Lander-Kruglyak 1995

Supplementary Table 8. Comparison of Polygenic risk score estimated from PGC SCZ GWAS summary statistic between BP1 individuals and controls. P-values are computed using linear and logistic regression models by taking into account relatedness; BP: coefficients and p-values for BP1 status; AdMix: coefficients and p-values for global admixture proportions of European ancestry; LM: Linear model; LR: Logistic regression; QNPRS: quantile-normalized polygenic risk scores.

GWAS Threshold	NSNPs	LM Beta (BP)	LM P-value (BP)	LM Beta (AdMix)	LM P-value (AdMix)	LR Log OR (QNPRS)	LR P-value (QNPRS)	OR for 1-unit increase in QNPRS	LR Log OR (AdMix)	LR P-value (AdMix)
0.01	24346	0.13	0.1247	-4.98	3.88E-13	0.22	0.0910	1.247	5.34	1.80E-04
0.001	6141	0.07	0.4552	-4.17	1.14E-08	0.14	0.2698	1.146	4.79	4.13E-04
0.0001	1874	0.02	0.8166	-3.10	4.68E-05	0.06	0.6361	1.057	4.43	7.30E-04
0.000001	337	0.00	0.9867	-0.40	6.27E-01	-0.02	0.8277	0.976	4.27	7.85E-04
0.00000005	143	-0.01	0.8765	0.36	6.67E-01	-0.07	0.5514	0.936	4.28	7.48E-04

Supplementary Table 9. Burden analysis of rare CNVs. CNV Type denotes the category of CNVs tested; No. of CNVs: the total number of CNVs detected; No. of BP1 CNVs: the number of CNVs affecting BP1 genes; BP: coefficients and p-values for BP1 status; AdMix: coefficients and p-values for global admixture proportions of European ancestry; LM: Linear model; LR: Logistic regression; QNBurden: quantile-normalized burden score.

CNV Type	No. of CNVs	No. of BP1 CNVs	LM Beta (BP)	LM P-value (BP)	LM Beta (AdMix)	LM P-value (AdMix)	LR Log OR (QNBurden)	LR P-value (QNBurden)	OR for 1-unit increase in QNBurden	LR Log OR (AdMix)	LR P-value (AdMix)
SNP DEL+DUP	2186	705	0.29	0.0130	-0.21	7.30E-01	0.29	0.0180	1.342	4.64	1.02E-03
SNP DUP	873	368	-0.05	0.6700	-0.57	3.50E-01	-0.05	0.6600	0.949	4.58	1.16E-03
SNP DEL	1313	337	0.35	0.0022	0.37	5.40E-01	0.36	0.0038	1.440	4.48	1.73E-03
WGS DEL	4436	737	0.27	0.0220	-0.23	7.00E-01	0.27	0.0330	1.305	4.37	1.59E-03
WGS DEL+10SNPs	1511	363	0.32	0.0061	-0.36	5.40E-01	0.33	0.0087	1.392	4.43	1.51E-03

Supplementary Table 10. The number of rare variants with genotype missing rate < 5% and at least two affected individuals sharing the variant in a family.

Family	SNV	CNV
CO10	241	17
CO14	386	16
CO15	289	21
CO18	278	28
CO23	499	27
CO25	451	32
CO27	314	24
CO4	400	25
CO7	1152	64
CO8	218	14
CR001	206	14
CR004	718	41
CR006	128	17
CR007	44	2
CR008	295	26
CR009	487	40
CR010	204	15
CR012	218	22
CR014	282	22
CR016	273	20
CR201	1782	101
CR277	131	16

Supplementary Table 11. Segregation results of rare SNVs for three different tests. #RV: the number of rare variants in a gene. All BP positions are on build 37, hg19.

	CHR	BP	Gene	FamilyID	#RV	P-value
Gene-level	2	98271420	*ACTR1B*	CR201	1	0.000518
	2	205409707	*PARD3B*	CO23	1	0.001046
	6	24803787	*FAM65B*	CO23	1	0.001338
	12	49418435	*MLL2*	CO7,CR004,CR012,CR016	4	0.0015042
	12	53876078	*MAP3K12*	CR001,CR004	1	0.00151346
	4	128553137	*INTU*	CR004,CR201,CR277	1	0.0017158
	11	118063479	*AMICA1*	CR006	1	0.001958
	17	36921648	*PIP4K2B*	CO23,CR201	1	0.00220019
	1	223281759	*TLR5*	CO7,CR010	2	0.00247749
	6	117072450	*FAM162B*	CO14,CO7,CR001	1	0.00267978
Variant-level	2	98275876	*ACTR1B*	CR201	1	0.000518
	1	228482059	*OBSCN*	CO7	1	0.000818
	6	90371215	*MDN1*	CO23,CR004	1	0.0009749
	2	196681435	*DNAH7*	CO23	1	0.001046
	2	205986458	*PARD3B*	CO23	1	0.001046
	12	49434801	*MLL2*	CR004	1	0.001241
	6	24848287	*FAM65B*	CO23	1	0.001338
	12	53876573	*MAP3K12*	CR001,CR004	1	0.00151346
	4	128626819	*INTU*	CR004,CR201,CR277	1	0.0017158
	11	118067537	*AMICA1*	CR006	1	0.001958
Family-level	6	117083172	*FAM162B*	CO7	1	0.000377
	2	98275876	*ACTR1B*	CR201	1	0.000518
	1	228482059	*OBSCN*	CO7	1	0.000818
	3	140401440	*TRIM42*	CR012	1	0.000972
	2	196681435	*DNAH7*	CO23	1	0.001046
	2	205986458	*PARD3B*	CO23	1	0.001046
	12	49434801	*MLL2*	CR004	1	0.001241
	12	53876573	*MAP3K12*	CR004	1	0.001241
	6	24848287	*FAM65B*	CO23	1	0.001338
	17	36935737	*PIP4K2B*	CR201	1	0.001602

Supplementary Table 12. Segregation results of rare CNVs for three different tests. #RV: the number of rare variants in a gene. All BP positions are on build 37, hg19.

	CHR	BP	Gene	FamilyID	#RV	P-value
Gene-level	5	32161816	*GOLPH3*	CO27	1	0.000789
	21	40594759	*BRWD1*	CO7	1	0.008174
	7	1495145	*MICALL2*	CO23,CR201	1	0.014247
	18	72672166	*ZNF407*	CR004	1	0.014854
	3	147155005	*ZIC1*	CO15,CO7	1	0.025583
	3	183433723	*YEATS2*	CR010	1	0.031282
	10	18670518	*CACNB2*	CO18	1	0.031311
	1	1223477	*SCNN1D*	CO4	1	0.034854
	3	52614424	*PBRM1*	CO14,CO18,CR008	2	0.040371
	22	43974737	*EFCAB6*	CO23,CO7	2	0.040386
Variant-level	5	32161816	*GOLPH3*	CO27	1	0.000789
	21	40594759	*BRWD1*	CO7	1	0.008174
	22	43974737	*EFCAB6*	CO23	1	0.012922
	7	1495145	*MICALL2*	CO23,CR201	1	0.014247
	18	72672166	*ZNF407*	CR004	1	0.014854
	3	147155005	*ZIC1*	CO15,CO7	1	0.025583
	3	183433723	*YEATS2*	CR010	1	0.031282
	10	18670518	*CACNB2*	CO18	1	0.031311
	12	99818192	*ANKS1B*	CO27	1	0.032181
	1	1223649	*SCNN1D*	CO4	1	0.034854
Family-level	5	32161816	*GOLPH3*	CO27	1	0.000789
	21	40594759	*BRWD1*	CO7	1	0.008174
	22	43974737	*EFCAB6*	CO23	1	0.012922
	18	72672166	*ZNF407*	CR004	1	0.014854
	3	147155005	*ZIC1*	CO15	1	0.015621
	6	16259314	*GMPR*	CR004	1	0.031155
	6	159419156	*RSPH3*	CO7	1	0.031242
	3	183433723	*YEATS2*	CR010	1	0.031282
	3	52669549	*PBRM1*	CO18	1	0.031311
	3	52669566	*PBRM1*	CO18	1	0.031311

Supplementary Table 13. The distribution of a segregation statistic and its p-value for the rare variant (rs141238033) present in the top gene (*ACTR1B*) from the SNV segregation analysis. This variant appears in family CR201 from founder ID 48. The observed Srare statistic is 46, which has p-value of 5.18E-04.

Statistic	Count	Pvalue
36	28	1
37	906	0.999972
38	8764	0.999066
39	58461	0.990302
40	180615	0.931841
41	429062	0.751226
42	219269	0.322164
43	78690	0.102895
44	19987	0.024205
45	3700	0.004218
46	464	5.18E-04
47	52	5.40E-05
48	2	2.00E-06

Supplementary Table 14. The distribution of a segregation statistic and its p-value for the rare variant (DEL_P0095_217) present in the top gene (*GOLPH3*) from the CNV segregation analysis. This variant appears in family CO27 from founder ID 18. The observed Srare statistic is 14, which has p-value of 7.89E-04.

Statistic	Count	Pvalue
3	9	1
4	480	0.999991
5	4665	0.999511
6	20958	0.994846
7	58634	0.973888
8	108181	0.915254
9	139165	0.807073
10	445765	0.667908
11	161155	0.222143
12	48242	0.060988
13	11957	1.27E-02
14	731	7.89E-04
15	58	5.80E-05

Supplementary Text

Ascertainment and Phenotypic Assessment of Study Sample

The study sample consisted of members of 26 pedigrees, 15 from CR and 11 from CO, each ascertained based on multiple members who had previously been clinically diagnosed with BP1. Our group has studied three of the CR families[1, 2] and several of the CO families[3] in past linkage studies of BP1, however the composition of the families changed somewhat as we recruited new individuals. We increased the number of families for study by recruiting family members of BP1 individuals individually recruited for population-level mapping studies[4, 5]. We expanded each family by systematically evaluating first degree relatives of the BP1 proband, and included other branches as we encountered individuals with suspected BP1. The ascertainment and phenotyping strategy employed in expansion of these pedigrees was previously reported in Fears *et al.*[6]. Due to the way the pedigree was expanded, with an emphasis on collecting equivalent numbers of BP1 individuals and their unaffected relatives, and with systematic preferred expansion of branches with BP1 individuals, the BP1 phenotype itself is not heritable in this pedigree collection.

Diagnostic interviews were conducted using the Mini International Neuropsychiatric Interview (M.I.N.I)[7] and a Spanish version of the DIGS; the CO clinical team developed this translated and validated[8] version and made it publicly available at https://www.nimhgenetics.org/interviews/digs_3.0_b/. Interviews were performed by bilingual psychologists or psychiatrists extensively trained in the use of these instruments. Among CR individuals newly recruited for this study, only those who answered positively to M.I.N.I questions related to mood or psychotic symptoms were targeted for the complete DIGS interview. All available CO family members received a DIGS interview. Six research psychiatrists or psychologists, at the University of California, San Francisco (VR), University of California, Los Angeles (CEB, JGF, JM), University of Antioquia (CLJ), and Rutgers University (JE), who are experts in the diagnosis of mood disorders reviewed all available clinical material for the cases (DIGS interview, medical records, hospital notes), with at least one expert rater reviewing each case. Prior to beginning Best Estimate (BE) procedures each diagnostician established reliability (diagnostic agreement of 90% or better) with the Chair of the BE group (VR). Individuals designated as BP1 had a BE diagnosis of BP1, unipolar mania, or schizoaffective disorder, bipolar type. Control individuals were those who went through the complete psychiatric evaluation and were found to have no mental illness, as well as those who answered negatively to all M.I.N.I.[7] questions related to mood or psychotic symptoms, and were $\geq$60 years of age.

Results of quality of variant calling and QC of WGS data

Variant calling: Illumina performed WGS of 454 individuals using HiSeq 2000 with 36x overall coverage. Illumina performed initial variant calling using their internal variant caller, CASAVA, to obtain SNV calls in the VCF format. BAM files were also made available after the CASAVA variant calling pipeline. To improve accuracy of variant calls, we re-aligned and re-called the WGS data using the GATK best practices[9]. We converted the original BAM files to FASTQ files,

and used the Churchill pipeline[10], which is an efficient and scalable implementation of the GATK best practices pipeline. We used the HaplotypeCaller of GATK (version 3.5-0-g36282e4). First, each individual was called separately to generate a GVCF file, and all individuals were joint-called using the GenotypeGVCFs tool in GATK. The variant calling was performed in the high performance cluster at UCLA called the Hoffman2 cluster. Variant calling resulted in 26.15 million (M) SNVs on autosomes and the X chromosome.

Individual-level QC of WGS Data: Before checking sequencing quality of each individual, we first removed variants that failed Variant Quality Score Recalibration (VQSR) in the GATK pipeline and we set each genotype whose genotype quality (GQ) score was ≤ 20 to missing. Additionally, all multi-allelic SNVs (0.44% of all SNVs after the VQSR filter) were excluded. In the remaining variants, for each sequenced individual we assessed genotype missing rate, the number of singletons and WGS genotype concordance with array genotypes, verified agreement between reported sex and sex determined from X chromosome markers, and compared empirical estimates of kinship with theoretical estimates. We also performed principal component analysis (PCA) using 1000 Genomes (1KG) Phase 3 as a reference panel[11]. We used EIGENSTRAT[12] for PCA, and included only founders from WGS data, and used independent common SNVs present in both BP1 WGS and 1KG Phase 3. We identified three individuals with poor sequencing quality who had high genotype missing rate (> 5%) or an excessive number of singletons and subsequently excluded the WGS data of these individuals. In comparison with array genotype data, we discovered two possible sample mix-ups, and WGS data from these samples were excluded. In the remaining subjects, all empirically derived kinship coefficients were consistent with theoretical kinship and genetic sex agreed with reported sex. In PCA plots, all founders from BP WGS were mapped close to Admixed American (AMR), especially in close proximity to Colombians from Medellin, Colombia (CLM).[11]

Variant-level QC of WGS Data: In doing variant-site QC, rather than using fixed thresholds for missingness, HWE, etc. to filter out poor quality variant sites, we used logistic regression to predict the probability of variant sites being of good or poor quality, with prediction based on mean and standard deviation of variant sequencing depth and genotype quality and a fraction of individuals meeting certain thresholds for depth and quality. We trained the regression model on a set of variants deemed to be of obvious good and poor quality using several QC measures such as genotype missing rate, HWE p-value, and the number of Mendelian errors. We assessed the accuracy of the model using cross validation (see the next section for details).

Genotype Refinement Using Pedigree-Aware Variant Calling Algorithm: We used the pedigree-aware variant calling method Polymutt[13] to refine genotypes of variant sites that passed QC. This method takes a VCF file with genotype likelihood generated from GATK and pedigree structure as input and generates a VCF file that refines genotype calls based on their likelihood and pedigree structure. To use Polymutt, some of large pedigrees had to be trimmed to remove inbreeding loops and reduce pedigree complexity. Among the 20,907,280 SNVs with good quality after applying the classifier, the number of Mendelian Errors (MEs) among all trios and pairs before application of Polymutt[1313131313](13)(Li et al., 2012) was 157,028, whereas the number of MEs after application of Polymutt was 266. Polymutt mostly changed genotypes

causing MEs; it changed 36.57% of genotypes that caused MEs while it changed 0.02% of genotypes that did not cause MEs. Genotypes changed by Polymutt had much lower GQ and DP than genotypes unchanged by Polymutt on average.

Imputation of Sequenced Sites into Genotyped Individuals: The 454 individuals in 22 of the 26 CO/CR families who underwent WGS were selected with the goal of imputing the rest of 334 family members who were genotyped on the Omni 2.5 chip, but not directly sequenced (Supplementary Figure 1). We used the imputation software GIGI[14], an approach designed to impute genotypes on large extended pedigrees. First, we obtained a genetic map of variant sites using the Rutgers genetic map interpolator[15]. We then used MORGAN[16] to obtain pedigree IVs based on independent common SNP array genotype data of individuals in 22 families with WGS data. GIGI used these IVs to impute WGS variants identified in the directly sequenced individuals into the pedigree members with only SNP genotype data. GIGI also imputed sequence data for pedigree members who were neither sequenced nor genotyped in the Omni 2.5 chip. GIGI imputed each family separately, and we further divided each chromosome into 10 Mb intervals to perform efficient imputation by utilizing parallelization in the high performance cluster. After imputation, GIGI generated the probability of each imputed genotype and we used the threshold-based calling with the default threshold to call genotypes for the rare variant burden analysis while we used the most likely genotype calls for the rare variant segregation analysis. For the threshold-based calling approach, only genotypes with probability in excess of 80% were called, genotypes that did not meet this threshold were set to missing. After the GIGI imputation, there were 782 individuals who were either sequenced or imputed with the high quality in 22 pedigrees. Among them, 190 are BP1 and 130 are controls.

Measuring accuracy of imputation: One measure to assess the imputation quality is the number of MEs after imputation. Considering all SNVs, we observed no MEs with the threshold-based calling and 239 MEs with the most likely genotype calls. Another measure is the missing rate of imputed genotypes in individuals without WGS data, as the higher missing rate indicates the lower imputation quality. One subject among the 334 imputed individuals had missing rate > 10% and was excluded (Supplementary Figure 1). For SNVs, 37.23% of common variants (MAF > 3%) and 0.37% of rare variants (MAF < 3%) had missing rate > 10% (computed from 334 imputed subjects). Another measure for imputation quality is to check genotype concordance between SNV genotype data and imputed data for SNVs. A majority of SNVs in the Omni 2.5 chip were present in WGS data and imputed during the GIGI imputation process. Excluding the individual who had the high imputation missing rate, all individuals with the SNV genotype data had genotype concordance rate > 97.93% between the Omni 2.5 chip and GIGI imputation data for non-missing calls in both datasets. Among those individuals, 80.48% had genotype missing rate < 10% for imputed data on SNVs present in Omni 2.5 chip. The genotype concordance rate between SNV and imputed data and the genotype missing rate of imputation had high negative correlation as expected (r=-0.877) (Supplementary Figure 2).

Building a logistic regression model to predict the quality of variants

In genetic studies, a few quality control (QC) measures are assessed to filter out variants with poor quality. For example, we may discard a variant if its genotype missing rate among genotyped individuals is > 10% or its Hardy Weinberg equilibrium (HWE) p-value is < 1E-7. These variants may represent sequencing or genotyping errors and may cause spurious findings. It is not, however, straightforward to choose the right threshold values for the QC measures, and they are often chosen arbitrarily. This approach may include variants with poor quality in analysis or may exclude variants with good quality.

In this paper, we aimed to build a classifier based on a logistic regression model to predict whether a variant has good or poor quality using its sequencing quality measures. First, we chose a set of variants that clearly have good and poor quality to train the classifier. SNV variants with good quality needed to meet all following criteria: 0 Mendelian errors (MEs) among all trios and pairs, genotype missing rate < 0.5%, HWE p-value > 0.01, and 0 or 1 discordant genotype to Omni 2.5 chip if variants were typed in Omni 2.5 chip. There were 20,314,194 SNV variants (94.10% of total variants) with good quality that satisfied all these criteria. Variants have clearly poor quality if they meet all of the following criteria depending on their minor allele frequency (MAF). For common variants (MAF > 3%), # of MEs > 5 and genotype missing rate > 3% and HWE p-value < 5E-4. For rare variants (MAF < 3%), # MEs > 3 and genotype missing rate > 2% and HWE p-value < 5E-3. If variants were typed in Omni 2.5 chip, they needed to have more than 6 discordant genotypes to Omni 2.5 chip to be considered to have poor quality. There were 4,957 SNVs (0.02% of total variants) with poor quality using these criteria, and 1,268,616 SNVs (5.88% of total variants) with uncertain quality. Our aim is to predict whether the 1,268,616 SNVs have good or poor quality using our classifier.

We used the following six sequencing quality measures of those variants with good and poor quality to train the classifier: 1) average sequencing depth (DP), 2) average genotype quality (GQ), 3) standard deviation of DP, 4) standard deviation of GQ, 5) a fraction of individuals whose DP is < 34 for SNVs, and 6) a fraction of individuals whose GQ is < 99 for SNVs. The rationale for using these quality measures is that variants with better quality are likely to have higher average DP and GP, lower SD of DP and GQ, and the lower fraction of individuals with DP and GQ < the thresholds. The thresholds (34 and 32 for DP and 99 and 81 for GQ) were chosen because they corresponded to the first quartile of the distribution of DP and GQ of genotypes that do not cause Mendelian errors for SNVs and are consistent with genotypes in Omni 2.5 chip for SNVs on chromosome 1. These sequencing quality measures were computed over the 449 subjects after removing five individuals with poor quality sequencing and sample mix-up.

To determine how accurately these six sequencing quality measures predict the quality of variants, we performed 10-fold cross validation in which we divided a set of variants with good and poor quality into 10 subsets. We trained a classifier using 9 subsets and tested it on the remaining set. We measured the accuracy of the classifier, which is the fraction of correct classifications on the 1/10 of variants. We repeated this procedure for all 10 subsets and measured accuracy and regression coefficients of each subset. Because the number of variants with good quality is much greater than that with poor quality (20M vs. 4,957 variants for SNVs), we randomly chose the same number of variants with good quality as the number of variants

with poor quality. We found that the average accuracy of our classifier using the 10-fold cross validation is 99.17% for SNVs.

We built the classifier by using the average regression coefficients estimated from the 10-fold cross validation and applied it to the set of variants with uncertain quality. We found, however, that some of those variants had extremely poor quality measures on one or two criteria, and we decided to mark them as outliers that would be considered as variants with poor quality. A variant was an outlier if it failed one of the following QC; # of discordant genotypes to Omni 2.5 chip > 20 for SNVs typed in Omni 2.5 chip, # of MEs > 10 and > 8 for common and rare variants, respectively, genotype missing rate > 10% and 8% for common and rare variants, respectively, and HWE p-value < 1E-8 and < 0.001 for common and rare variants, respectively. The number of outlier variants was 406,840 (32% among variants with uncertain quality) for SNVs. We applied the classifier to the rest of variants with uncertain quality to obtain the probability of them having good quality, and we considered them as variants with good quality if this probability was > 80%. We found that 68.82% of SNVs with uncertain quality were predicted to have good quality. In the end, there were 20,907,280 SNVs with good quality (96.85% of total SNVs) and 680,477 SNVs with poor quality (3.15%). Summary QC statistics for these 20.91M SNVs can be found in Supplementary Table 1.

CNV calling using genotyping arrays

SNP genotyping was performed in three separate batches. In order to obtain the most accurate intensity measures and reduce inter-batch variability, we first produced an initial set of intensity measures and genotyping calls using the canonical cluster file (*.egt) in Beeline 2.0 (Illumina). We excluded all assays with an LRRSD > 0.30 and a genotype call rate < 98%, and generated custom cluster files generated for each genotyping batch individually in GenomeStudio 2.0 (Illumina). These cluster files were then used to generate new LRR and BAF values. We then restricted our analysis to the 2,364,179 SNP assays common across all versions of the Omni2.5 arrays and ran two separate CNV calling algorithms, PennCNV[17] and QuantiSNP.[18] PennCNV was run as recommended by the developer with waviness correction[19] and a custom *.pfb file generated from all samples. QuantiSNP 2.0 was run using default settings including GC-based correction of LRR. Calls from both algorithms were compared on the sample level, and retained only if they were called by both algorithms (based on any overlap) and of the same relative type (gain or loss). For these overlapping calls, we retained the CNV boundaries as defined by PennCNV.

We removed intensity outliers (mean +/- 3 SD or by visual inspection) based on three different metrics extracted from QC logs produced by PennCNV (Supplementary Figure 3): LRR standard deviation (LRR_SD), BAF standard deviation (BAF_SD), and waviness factor (WF). Samples were excluded if they had an LRR_SD > 0.21, a BAF_SD > 0.054 or |WF| > 0.02. We then performed post-segmentation cleaning of fragmented CNV calls using PennCNV's "clean_cnv.pl" script. Adjacent calls of the same type were merged if the number of intervening markers was less than 20% of the combined segment. We removed calls in regions known to produce spurious CNV calls. Calls were removed if they spanned or were contained within centromeric regions

(+500K), or if they overlapped with telomeric regions (+100K), VDJ recombination regions, or segmental duplications by more than 50% of their length. After filtering for rare events (see Methods) spanned by a minimum of 10 SNPs and > 5kb in length, we also removed samples with excessive CNV load, both in terms of the number of CNV segments (n > 50) and total autosomal CNV length (>5Mb).

CNV calling using WGS

We used GenomeSTRiP v2.00.1747[20] to detect and genotype CNVs across all samples with WGS. Alignment files were first preprocessed using a reference metadata bundle adapted from the Broad's HG19 version (ftp://ftp.broadinstitute.org/pub/svtoolkit/reference_metadata_bundles) using a 100bp alignability mask. Our aim was to impute CNVs detected in the subset of samples with WGS. Therefore, we focused on deletions only, since deletions are most reliably imputed[20] and also because our imputation method relies on the availability of discrete genotype calls. To discover deletions, we ran the SVDiscovery pipeline script to in two different batches to discover variants of different size ranges: 100bp-100kbp and 100kbp-10Mb. Sites from both discovery runs were filtered using recommended parameters established by the Genome of the Netherlands Consortium.[21] Discovery sites with an alpha-satellite fraction of >0.9 were also excluded. We excluded outlying samples with an excessive number of autosomal discovery CNVs (Supplementary Figure 4), and genotyped all remaining samples using read-depth on a total of 10,609 putative deletions events.

Inspection of the resultant genotypes in available trios (n=68) demonstrated consistently low levels of Mendelian inconsistencies across these candidate loci (0.0026±0.00067, Supplementary Figure 4). All genotyped sites were further refined based genotype calls using the following criteria: (i) duplicate sites with a 50% overlap and discordance LOD score greater than zero, (ii) non-variant sites without confident (GSNONVARSCORE ≥ 13) non-reference genotype calls, (iii) sites with cluster means deviating from expected values (GSM1 < 0.5 or > 2.0), and (iv) sites with poor cluster separation (GSCLUSTERSEP ≤ 2). Using an Intensity Rank Sum test on available SNP-array intensity data, we estimated a genome-wide FDR for the entire callset ranging from 0.04 (all deletions) decreasing to 0.018 (deletions ≥ 5kb) and lower, depending on the size-cutoff used.

Prior to performing imputation, we removed all monomorphic CNVs and CNVs with a genotype missing rate > 5% after setting Mendelian inconsistent genotypes to missing. We also removed CNVs failing within VDJ recombination regions. We then imputed CNVs across all samples using the same imputation pipeline for SNVs. We restricted our final callset to the set of samples with SNP genotyping data and kept only samples that were also well-imputed for SNVs.

We retained well-imputed CNVs using the threshold-based calling approach where a genotype with the highest probability needs to be > 80% and used a custom Perl scripts to convert our imputed callset to PLINK's native CNV format and annotate imputed CNV calls for their overlap with the Omni2.5 array. To make our callset comparable to 1000 Genomes data, we also removed all CNVs that had <200bp of uniquely-alignable sequence as defined by the 35-mer

alignability mask used by Phase 3 of the 1000 Genomes Project. To completely remove redundancy at the sample level (which could bias subsequent burden analysis), we also combined all strictly overlapping CNV calls within the same individual by taking the union of segment boundaries. We then used frequency information defined by 1000 Genomes Phase 3 Structural variant callset[22] to define a final set rare, imputed CNVs as described in the Main Methods.

Linkage analysis

Overview: Several of the 26 pedigrees are large and complex, with multiple inbreeding or marriage loops. Such pedigrees pose a significant challenge to analysis, as standard multipoint approaches that employ the Lander-Green algorithm fail in this setting. Possible solutions would include trimming and cutting pedigrees into smaller units, however we found that applying this approach in these pedigrees led to a reduced capability to resolve phase, and therefore resulted in substantially reduced power. We also considered MCMC approaches[23], however given the density of markers in a genome-wide setting, such approaches are exceedingly slow, and we found it difficult to evaluate if we had reached convergence. We evaluated linkage in our pedigrees using a two-point parametric linkage on both SNPs and multi-allelic STRs identified from the WGS data using LobSTR[24], as well as a non-parametric method proposed specifically for use in large, complex pedigrees[25].

Quality control of microarray data: A subset of samples was repeated in each batch to enable concordance checks. A total of 2,026,257 SNPs were polymorphic and passed all QC procedures, including the evaluation of call rate, testing for Hardy Weinberg equilibrium, and Mendelian error. For linkage studies we used 99,446 SNPs with MAF>0.35, that had been LD-pruned to have $r^2<0.5$. During QC procedures, allele frequency calculations, calculations of HWE, and estimates of LD were performed using only unrelated (founder) individuals. Further pedigree-wide Mendel checks were performed on the set of 99,446 SNPs used in linkage analysis; 0.7% of markers had 1 or more errors in Mendelian inheritance in these additional checks and all data for the marker in the family that generated the error was set to missing. Individual-level QC checks included verifying the pedigree structure by comparing theoretical kinship with empirical estimates, assessing missing rate, and verifying that the genetic sex agreed with the reported sex.

Identification and QC of STRs: We detected STRs with the lobSTR software[24] (version 4.0.0) that uses sequencing data to call STRs. The BAM files aligned with BWA-MEM that were generated during the variant calling process were used as input to lobSTR, which then generated VCF files for STR loci. To measure the accuracy of STR calls from the lobSTR software, we compared lobSTR STR calls to STR data previously collected for 19 individuals in one family; STRs were detected with electrophoresis. The previous STR data had 367 genome-wide STRs. Based on the filtering script that the lobSTR software provides, we developed different filtering strategies. For each filter, we measured the number of STRs passing the filter, the genotype concordance between the lobSTR STR calls and the previous STR data, and also the number of Mendelian inconsistencies using PedCheck software[26]. We then identified the filter that achieves greater

than 95% genotype concordance between the two STR calls and fewer than 0.1 Mendelian inconsistencies per STR. This filter removed 1) monomorphic STRs, 2) STRs with repeats of one nucleotide, 3) STRs with ambiguous repeating nucleotides, 4) STRs with call rate < 95%, coverage < 5x, or Q-score < 0.95. After applying the filter to lobSTR calls detected in all families, we retained 86,601 high-quality STR calls. Among them, 37,472 STRs (43.27%) are multi-allelic with more than two alleles.

Two-point Parametric Linkage Analysis: We used the software Mendel[27] to estimate allele frequencies in the 26 pedigrees, performing these estimates separately for the CR and CO samples, and accounting for pedigree relationships. Two-point parametric linkage with heterogeneity was done in Mendel for autosomes, the very large pedigree CR201 had to be broken into four non-overlapping sections in order to do this analysis. Our parametric model used disease frequency to be 0.003, and penetrance parameters: P(BP1|DD)=0.9, P(BP1|DN)=0.81 and P(BP1|NN)=0.01; where "D" represents the disease allele and "N" represents the normal allele. Individuals are considered either affected with BP1 or phenotype unknown, that is, we designate no individuals as controls. This is the same model employed in McInnes et al.[1] and Herzberg et al.[3]

The null hypothesis of no linkage was evaluated using the LOD (logarithm of odds) score, which is the log 10 of the ratio of the likelihood of linkage, given the estimated recombination fraction, to the likelihood given the null value for the recombination fraction (0.5). Traditionally, a LOD score of 3 (p=0.0001) was considered significant linkage, however Lander & Kruglyak[28] suggested LOD=3.3 (p=4.9E-05) was a more appropriate threshold. The HLOD (LOD with heterogeneity), tests 2 parameters: the recombination fraction and proportion of linked pedigrees. The HLOD score has traditionally been evaluated using a combination of chi-square distributions with 1 and 2 degrees of freedom[29]. By this metric, an HLOD of 3.6-3.7 corresponds to p~0.0001; using the Lander and Kruglyak p-value threshold of 4.9E-05 corresponds to an HLOD of 4.1.

Non-parametric Linkage Analysis: We used Rapid[25] for non-parametric analysis of allele-sharing of SNPs among BP1 individuals in our pedigrees. Rapid is designed for use in large complex pedigrees, where estimation of identity by descent (IBD) can be computationally demanding. Rapid evaluates significance using an approximation to the empiric p-value, which Abney et al.[25] states is conservative. After the first genome-wide scan to identify promising results using the approximation, we re-analyzed markers with –log10(P) > 3.5 with 250,000 simulations to obtain a true empiric p-value. Markers with p<4.9e-05 were then evaluated with 1,000,000 simulations to obtain a more accurate p-value.

Results: Four microarray SNPs displayed HLODs exceeding 4.1 (Supplementary Figure 6, Supplementary Table 5); two SNPs on 2q33 and 2q36 (kgp5681631 at 206.8 Mb and kgp10402924 at 228.3 Mb, with HLODs of 4.71 and 4.50, respectively) and two SNPs on 16q23 (kgp2684721 at 79.5 Mb and rs8058020 at 83.7 Mb, with HLODs of 4.13 and 4.59, respectively). There was no heterogeneity for the linkage results on 2q33 and 2q36, and on 16q23 the estimate of the proportion of linked families (denoted as *a*) was 0.90. The HLOD for two STRs

exceeded the threshold of 4.1, one on 1q44 (247.1 Mb, HLOD=5.03, a = 0.72), and one on 15q14 (35.3 Mb, HLOD=4.32, a = 0.62). While these results at five loci are suggestive, our simulation studies indicate that the evidence in favor of linkage is not as strong as it might appear. We carried out simulations under the null hypothesis of no linkage to BP1 anywhere in the genome using two different strategies. In one case, we kept fixed the phenotype information and used gene dropping to generate genotypes for markers with allele frequencies corresponding to the one observed in our sample; in the other case, we kept the observed genotypes and permuted the phenotype values (with appropriate consideration of family structure). Considering all sets of simulations, HLODs of 4.1 or greater were seen in 80% of replicate genome screens, suggesting that, in these pedigrees, 4.1 is likely not an appropriate threshold for declaring genome-wide significant linkage.

In the NPL analysis, we approximated the empiric p-values using Rapid, and 13 SNPs and 156 STRs resulted in –log10(P)>3.5. These SNPs and STRs were re-analyzed with 250,000 simulations to obtain a true empiric p-value, and among those, eight SNPs and one STR had p<4.9e-05, which corresponds to the HLOD threshold > 4.1. We re-analyzed those eight SNPs and one STR with 1,000,000 simulations, and empiric p-values from this round of simulations ranged from 2e-06 to 3.9e-05, including two SNPs with p=2e-06, one on 16q23 (at 77.7 Mb) and one SNP on 21q21 (at 23.9 Mb) (Supplementary Table 5).

Identification of genes for burden and segregation analyses for rare variants

We highlighted genes for burden and segregation analysis of rare variants from three sources: (1) genes in regions that demonstrated evidence of enrichment of BP1 heritability, as evaluated in the PGC BP1 GWAS data set (summary GWAS statistics); (2) genes near PGC BP1 GWAS peaks; (3) genes within 1Mb of our linkage peaks. We only consider genes where there is at least one deleterious SNV in our BP1 dataset.

We identified regions with enrichment of BP1 heritability using PGC GWAS summary statistics and 220 epigenetic profiles that originated from 100 individual cell types or tissues. The epigenetic annotation came from cell-type specific histone modification (ChIP-seq) data generated by the NIH Roadmap Epigenome Project[30]. H3K4me1, H3K4me3 and H3K9ac data were post-processed by Trynka et al.[31] The definition of annotations used in our analysis have been previously described.[32, 33] Briefly, peaks were called using MACS v.1.4. For each cell-type and specific histone mark, start and end position of identified regions (i.e. called peaks) were determined and together define an annotation. H3K27ac data were post-processed separately by Hnisz et al.[34], also using MACS v.1.4. The 220 cell type-specific annotations were then divided into 10 groups by taking a union of the cell type-specific annotation within each group, following Finucane et al.[33] Within all regions marked for a specific tissue group, we evaluated the possible enrichment of BP1 heritability using PGC BP1 GWAS summary statistics and Stratified LD Score Regression, a statistical method that partitions SNP-based h2 from GWAS summary statistics.[33] For tissue groups that demonstrated significant BP1 heritability in the PGC, we then highlighted for further analysis in our BP1 families all genes within regions

marked by the annotation process; that is, all genes overlapping intervals defined by start and end positions of identified regions (peaks).

For each cell-type group annotation, we first estimated partitioned LD scores using the ldsc.py --l2 function with MAF > 5%, a 1 centimorgan (cm) window, and the 1000 Genomes European reference panel (CEU) to estimate LD matched to the population of the PGC GWAS. We next ran sLDSR (ldsc.py --h2) for each cell-type group while accounting for the overall heritability that is distributed across 53 baseline annotations (baseline model), as recommended by the developers. If a cell-type group annotation is associated with increased h2, LD to SNPs in that annotation will increase the $\chi 2$ statistic of a SNP more than LD to SNPs outside of that cell-type annotation. To determine if this effect is significant, it estimates the contribution of that annotation to the per-SNP h2 while accounting for the baseline model. We evaluate the Z-scores of the contribution of each annotation, which is calculated using standard errors that are obtained via a block jackknife procedure.

We also specifically targeted genes near genome-wide significant association signals in the PGC BP1 GWAS. The PGC BP1 GWAS summary statistics were clumped in PLINK, using our BP1 genotype data as the LD reference (founder genotypes only). Clumps were formed in windows of 250 Kb and using an r^2 threshold of 0.1. Among the resultant clumps, if the lead SNV was genome-wide significantly associated to BP1 in the PGC data (p<5e-08), we determined the physical extent of the SNVs that were in the same clump as the lead SNP, and considered for further analysis all genes within such regions.

Lastly we targeted genes within 1 Mb of linkage peaks (both parametric and non-parametric) identified in our BP1 pedigrees. Linkage peaks were defined as parametric HLOD>4.1, or non-parametric p-value <4.9e-05.

Identifying founders who carried a rare variant in the segregation method

To detect F^{rv}, founders who introduced the rare variant into a family, we used the results of GIGI imputation. GIGI generated the probability of imputed genotypes for everyone in a family, even those who were not genotyped with the microarray (including all founders). Assuming bi-allelic variants, GIGI generated three probabilities for three genotypes (1/1, 1/2, and 2/2 where 1 and 2 are two alleles). We had two strategies to identify F^{rv}. In the first strategy, if the highest genotype probability among the three probabilities of a founder was > 0.8, we considered this founder to have a high-quality genotype. If all founders in a family had high-quality genotypes, we included this rare variant for analysis and identified F^{rv} who had a rare variant. We used the second strategy when all founders except a pair of top founders had high-quality genotypes. A pair of top founders was a couple who did not both have parents in the pedigree structure, and they were not usually sequenced or imputed well. In some cases, GIGI assigned about 0.5 probability of having a rare variant to both top founders. This indicated that a rare variant was inherited from one of the couples, but GIGI was not able to accurately identify which founder introduced the variant. Assuming that both top founders were neither sequenced nor imputed well, and they did not contribute to the S_{rare} statistic, the segregation p-value would be the

same regardless of which of the founders carried the rare variant. Hence, we randomly assigned the rare variant to one of the top founders in the second strategy. We ignored rare variants in which there was more than one pair of top founder in a family who did not have the high-quality genotypes as well as rare variants in which founders carried two alleles of a rare variant. We analyzed rare variants with low genotype missing rate (< 5%) that at least two affected individuals shared in a family as we were not interested in rare variants present in one or zero affected individuals.

Performance of the segregation method for rare SNVs and CNVs

To measure the performance of the segregation method for rare SNVs and CNVs, we generated simulation data following the simulation framework of Sul et al.[35]. We used a modified version of the wide pedigree structure in Sul et al.; the original wide pedigree structure had 30 individuals where there were two founders in the first generation, two founders and two nonfounders in the second generation, and 24 nonfounders in the third generation (see Figure S1 in Sul et al.). We doubled the number of nonfounders in this simulation to simulate a larger pedigree structure. We used COSI software to generate haplotypes of unrelated individuals assuming European ancestry and assigned them to founders such that only one founder had a rare variant. We then generated haplotypes of nonfounders using a gene dropping procedure from founders (refer to Sul et al. for detailed descriptions of simulation framework). To measure the false positive rate (FPR) of the segregation method, we generated the null simulation dataset by randomly assigning affected and unaffected status to individuals in pedigrees. To measure the power, affection status of each individual was determined with the following logistic regression model.

$$P(A=1) = \frac{exp(\beta_0 + X^T\beta)}{1 + exp(\beta_0 + X^T\beta)}$$

P(A=1) was the probability that individual A was affected, and $\beta_0 = \log\left(W/(1-W)\right)$ where W was the baseline prevalence, which is 20% in our simulation. X was the genotype vector where 1 means carrying a rare variant. $\beta = \log\left(OR\right)$ and we used odds ratio (OR) of 4, 6, 8, 10 and 12. We used large odds ratio to simulate the perfect segregation or nearly perfect segregation in a family. We generated 10,000 replicates for the false-positive simulation and 2,000 replicates for power simulation for each OR. We generated 20 families in each replicate, and the minimum number of affected individuals was 19 and the maximum number was 37 in each family. We performed 100,000 random IV sampling to estimate a p-value of each segregation, and we only tested family-level p-values in this simulation. We included only variants that were shared by at least two affected individuals in a family, which is consistent with how we tested segregation of rare variants for the CO/CR families. FPR was measured as a fraction of p-values < α = 0.1 and 0.05 among all p-values calculated (the number of families with a rare variant in all replicates), and the power was similarly calculated at α = 0.05. Results show that FRP was 0.088 at α = 0.1 and 0.039 at α = 0.05, which indicates our method had slightly lower FPR than expected. The power of our method was 0.54, 0.75, 0.84, 0.89, and 0.92 for OR of 4, 6, 8, 10 and 12, respectively. These results indicate that our method has the appropriate power to detect a rare variant with strong effect in a large family.

REFERENCES

1. McInnes LA, Escamilla MA, Service SK, Reus VI, Leon P, Silva S *et al.* A complete genome screen for genes predisposing to severe bipolar disorder in two Costa Rican pedigrees. *Proc Natl Acad Sci U S A* 1996; **93**(23)**:** 13060-13065.

2. Service S, Molina J, Deyoung J, Jawaheer D, Aldana I, Vu T *et al.* Results of a SNP genome screen in a large Costa Rican pedigree segregating for severe bipolar disorder. *Am J Med Genet B Neuropsychiatr Genet* 2006; **141B**(4)**:** 367-373.

3. Herzberg I, Jasinska A, Garcia J, Jawaheer D, Service S, Kremeyer B *et al.* Convergent linkage evidence from two Latin-American population isolates supports the presence of a susceptibility locus for bipolar disorder in 5q31-34. *Hum Mol Genet* 2006; **15**(21)**:** 3146-3153.

4. Ophoff RA, Escamilla MA, Service SK, Spesny M, Meshi DB, Poon W *et al.* Genomewide linkage disequilibrium mapping of severe bipolar disorder in a population isolate. *Am J Hum Genet* 2002; **71**(3)**:** 565-574.

5. Kremeyer B, Garcia J, Muller H, Burley MW, Herzberg I, Parra MV *et al.* Genome-wide linkage scan of bipolar disorder in a Colombian population isolate replicates Loci on chromosomes 7p21-22, 1p31, 16p12 and 21q21-22 and identifies a novel locus on chromosome 12q. *Hum Hered* 2010; **70**(4)**:** 255-268.

6. Fears SC, Service SK, Kremeyer B, Araya C, Araya X, Bejarano J *et al.* Multisystem component phenotypes of bipolar disorder for genetic investigations of extended pedigrees. *JAMA Psychiatry* 2014; **71**(4)**:** 375-387.

7. Sheehan DV, Lecrubier Y, Sheehan KH, Amorim P, Janavs J, Weiller E *et al.* The Mini-International Neuropsychiatric Interview (M.I.N.I.): the development and validation of a structured diagnostic psychiatric interview for DSM-IV and ICD-10. *J Clin Psychiatry* 1998; **59 Suppl 20:** 22-33;quiz 34-57.

8. Palacio CA, Garcia J, Arbelaez MP, Sanchez R, Aguirre B, Garces IC *et al.* [Validation of the Diagnostic Interview for Genetic Studies (DIGS) in Colombia]. *Biomedica* 2004; **24**(1)**:** 56-62.

9. DePristo MA, Banks E, Poplin R, Garimella KV, Maguire JR, Hartl C *et al.* A framework for variation discovery and genotyping using next-generation DNA sequencing data. *Nat Genet* 2011; **43**(5)**:** 491-498.

10. Kelly BJ, Fitch JR, Hu Y, Corsmeier DJ, Zhong H, Wetzel AN *et al.* Churchill: an ultra-fast, deterministic, highly scalable and balanced parallelization strategy for the discovery of human genetic variation in clinical and population-scale genomics. *Genome Biol* 2015; **16:** 6.

11. Genomes Project C, Auton A, Brooks LD, Durbin RM, Garrison EP, Kang HM *et al.* A global reference for human genetic variation. *Nature* 2015; **526**(7571)**:** 68-74.

12. Price AL, Patterson NJ, Plenge RM, Weinblatt ME, Shadick NA, Reich D. Principal components analysis corrects for stratification in genome-wide association studies. *Nat Genet* 2006; **38**(8)**:** 904-909.

13. Li B, Chen W, Zhan X, Busonero F, Sanna S, Sidore C *et al.* A likelihood-based framework for variant calling and de novo mutation detection in families. *PLoS Genet* 2012; **8**(10)**:** e1002944.

14. Cheung CY, Thompson EA, Wijsman EM. GIGI: an approach to effective imputation of dense genotypes on large pedigrees. *Am J Hum Genet* 2013; **92**(4)**:** 504-516.

15. Matise TC, Chen F, Chen W, De La Vega FM, Hansen M, He C *et al.* A second-generation combined linkage physical map of the human genome. *Genome Res* 2007; **17**(12)**:** 1783-1786.

16. Thompson E. The structure of genetic linkage data: from LIPED to 1M SNPs. *Hum Hered* 2011; **71**(2)**:** 86-96.

17. Wang K, Li M, Hadley D, Liu R, Glessner J, Grant SF *et al.* PennCNV: an integrated hidden Markov model designed for high-resolution copy number variation detection in whole-genome SNP genotyping data. *Genome Res* 2007; **17**(11)**:** 1665-1674.

18. Colella S, Yau C, Taylor JM, Mirza G, Butler H, Clouston P *et al.* QuantiSNP: an Objective Bayes Hidden-Markov Model to detect and accurately map copy number variation using SNP genotyping data. *Nucleic Acids Res* 2007; **35**(6)**:** 2013-2025.

19. Diskin SJ, Li M, Hou C, Yang S, Glessner J, Hakonarson H *et al.* Adjustment of genomic waves in signal intensities from whole-genome SNP genotyping platforms. *Nucleic Acids Res* 2008; **36**(19)**:** e126.

20. Handsaker RE, Van Doren V, Berman JR, Genovese G, Kashin S, Boettger LM *et al.* Large multiallelic copy number variations in humans. *Nat Genet* 2015; **47**(3)**:** 296-303.

21. Genome of the Netherlands C. Whole-genome sequence variation, population structure and demographic history of the Dutch population. *Nat Genet* 2014; **46**(8)**:** 818-825.

22. Sudmant PH, Rausch T, Gardner EJ, Handsaker RE, Abyzov A, Huddleston J *et al.* An integrated map of structural variation in 2,504 human genomes. *Nature* 2015; **526**(7571)**:** 75-81.

23. Garner C, McInnes LA, Service SK, Spesny M, Fournier E, Leon P *et al.* Linkage analysis of a complex pedigree with severe bipolar disorder, using a Markov chain Monte Carlo method. *Am J Hum Genet* 2001; **68**(4)**:** 1061-1064.

24. Gymrek M, Golan D, Rosset S, Erlich Y. lobSTR: A short tandem repeat profiler for personal genomes. *Genome Res* 2012; **22**(6)**:** 1154-1162.

25. Abney M. Identity-by-descent estimation and mapping of qualitative traits in large, complex pedigrees. *Genetics* 2008; **179**(3)**:** 1577-1590.

26. O'Connell JR, Weeks DE. PedCheck: a program for identification of genotype incompatibilities in linkage analysis. *Am J Hum Genet* 1998; **63**(1)**:** 259-266.

27. Lange K, Papp JC, Sinsheimer JS, Sripracha R, Zhou H, Sobel EM. Mendel: the Swiss army knife of genetic analysis programs. *Bioinformatics* 2013; **29**(12)**:** 1568-1570.

28. Lander E, Kruglyak L. Genetic dissection of complex traits: guidelines for interpreting and reporting linkage results. *Nat Genet* 1995; **11**(3)**:** 241-247.

29. Abreu PC, Hodge SE, Greenberg DA. Quantification of type I error probabilities for heterogeneity LOD scores. *Genet Epidemiol* 2002; **22**(2)**:** 156-169.

30. Bernstein BE, Stamatoyannopoulos JA, Costello JF, Ren B, Milosavljevic A, Meissner A *et al.* The NIH Roadmap Epigenomics Mapping Consortium. *Nat Biotechnol* 2010; **28**(10)**:** 1045-1048.

31. Trynka G, Sandor C, Han B, Xu H, Stranger BE, Liu XS *et al.* Chromatin marks identify critical cell types for fine mapping complex trait variants. *Nat Genet* 2013; **45**(2)**:** 124-130.

32. Gusev A, Lee SH, Trynka G, Finucane H, Vilhjalmsson BJ, Xu H *et al.* Partitioning heritability of regulatory and cell-type-specific variants across 11 common diseases. *Am J Hum Genet* 2014; **95**(5)**:** 535-552.

33. Finucane HK, Bulik-Sullivan B, Gusev A, Trynka G, Reshef Y, Loh PR *et al.* Partitioning heritability by functional annotation using genome-wide association summary statistics. *Nat Genet* 2015; **47**(11)**:** 1228-1235.

34. Hnisz D, Abraham BJ, Lee TI, Lau A, Saint-Andre V, Sigova AA *et al.* Super-enhancers in the control of cell identity and disease. *Cell* 2013; **155**(4)**:** 934-947.

35. Sul JH, Cade BE, Cho MH, Qiao D, Silverman EK, Redline S *et al.* Increasing Generality and Power of Rare-Variant Tests by Utilizing Extended Pedigrees. *Am J Hum Genet* 2016; **99**(4)**:** 846-859.
